# Benchmarking of T cell receptor-epitope predictors with ePytope-TCR

**DOI:** 10.1016/j.xgen.2025.100946

**Published:** 2025-07-07

**Authors:** Felix Drost, Anna Chernysheva, Mahmoud Albahah, Katharina Kocher, Kilian Schober, Benjamin Schubert

**Affiliations:** 1 Computational Health Center, Helmholtz Munich, 85764 Neuherberg, Germany; 2 School of Life Sciences Weihenstephan, Technical University of Munich, 85354 Freising, Germany; 3 Mikrobiologisches Institut - Klinische Mikrobiologie, Immunologie und Hygiene, Universitätsklinikum Erlangen und Friedrich-Alexander-Universität (FAU) Erlangen-Nürnberg, 91054 Erlangen, Germany; 4 FAU Profile Center Immunomedicine, FAU Erlangen-Nürnberg, 91054 Erlangen, Germany; 5 School of Computation, Information and Technology, Technical University of Munich, 85748 Garching bei München, Germany

**Keywords:** T cell receptor, TCR-epitope prediction, benchmarking, machine learning, deep learning, adaptive immunology, T cell immunology

## Abstract

Understanding the recognition of disease-derived epitopes through T cell receptors (TCRs) has the potential to serve as a stepping stone for the development of efficient immunotherapies and vaccines. While a plethora of sequence-based prediction methods for TCR-epitope binding exists, their pre-trained models have not been comparatively evaluated. To alleviate this shortcoming, we integrated 21 TCR-epitope prediction models into the immune-prediction framework ePytope, offering interoperable interfaces with standard TCR repertoire data formats. We showcase the applicability of ePytope-TCR by evaluating the performance of these publicly available prediction models on two challenging datasets. While novel predictors successfully predicted binding to frequently observed epitopes, all methods failed for less frequently observed epitopes. Further, we detected a strong bias in the prediction scores between different epitope classes. We envision this benchmark to guide researchers in their choice of a predictor and to accelerate the method development by defining standardized evaluation settings.

## Highlights

• ePytope-TCR integrated 21 binding predictors of T cell receptor toward epitopes
• The framework allowed evaluating the methods on two challenging benchmark datasets
• This test revealed biases and limited generalization for unknown target epitopes
• ePytope-TCR’s benchmark accelerates model selection and future method development

## Graphical abstract

**Figure:**
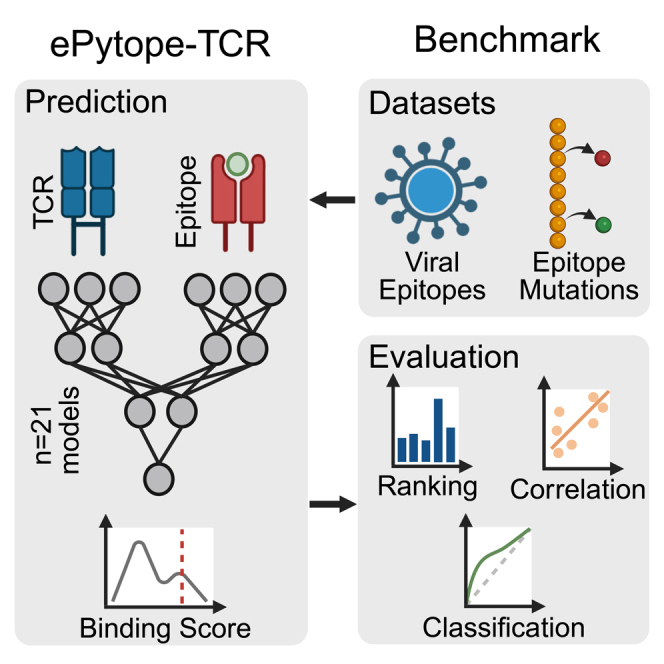

## Teaser

Drost et al. present ePytope-TCR, a unified framework of binding predictors for T cell receptors toward disease-derived epitopes. A comparative benchmark reveals biases and shortcomings of these predictors, thereby aiding in future method development and guiding applied researchers in their tool selection.

## Introduction

T cells play a fundamental role in the adaptive immune system by recognizing diseased cells through diverse T cell receptors (TCRs). Target cells present antigen-derived peptides—so-called epitopes—bound by the major histocompatibility complex (MHC) to the TCR. The TCR mainly interacts with the peptide by the complementary determining region 3 (CDR3) of its β-chain, while CDR1 and CDR2 ensure contact with the MHC.^1^ TCR specificity is crucial for understanding vaccine efficacy^2^ and developing immunotherapies against cancer^3^ and autoimmune diseases.^4^ By better understanding how TCRs recognize specific antigens, we can unlock new strategies for targeted treatments. Therefore, deciphering the TCR-epitope interaction was declared one of the nine Cancer Grand Challenges in 2023.^5^ Several solutions exist to discover pairs of TCRs and their cognate epitopes, such as high-throughput single-cell techniques for staining TCR repertoires with peptide-loaded multimers.^6^ However, these experiments are labor- and cost-intensive and typically require an initial set of target epitopes to test. *In silico* prediction methods could overcome these restrictions and provide antigen-specific TCR candidates.

In two seminal publications, Dash et al.^7^ and Glanville et al.^8^ demonstrated that the TCR sequence is indicative of epitope specificity when employed for pairwise distance calculation and clustering. Based on these findings, several approaches were developed to compare sequences between a query TCR and an atlas repertoire with known epitope specificity using string metrics,^7^^,^^9^ and later deep learning-based representations.^10^^,^^11^^,^^12^ While such distance- or embedding-based methods play a pivotal role in determining TCR specificity in applied research, these approaches require the target epitope to be contained in atlases such as the common databases IEDB,^13^ VDJdb,^14^ or McPAS-TCR,^15^ which may not be the case for newly arising epitopes from mutations or novel infectious diseases. With the increasing amount of publicly available TCR-epitope pairs, machine learning models have been trained to predict TCR specificity in two settings. In the first setting, the pre-defined epitopes are considered as categories, i.e., the models receive only the TCR as input and learn from a set of specific TCRs their sequence properties that are characteristic for the epitope class.^16^^,^^17^^,^^18^ As they do not incorporate the epitope sequence into their prediction, they only can be applied to the targets on which they were trained. Alternatively, models can encode the epitope sequences as an additional input to learn the TCR-epitope interaction.^19^^,^^20^ While such general predictors—in contrast to categorical models—also can be applied to unknown targets, this generalization typically results in a forfeit of predictive performance.^21^

While a plethora of prediction methods have been published, the comparative performance of their publicly available models remains unclear as they have been evaluated on different datasets and under differing settings. Recently, efforts have been made to evaluate methodological development by comparing predictors on standardized datasets^22^^,^^23^ notably by the ImmRep workshop.^24^^,^^25^^,^^26^ However, a thorough benchmark of available pre-trained models is missing to guide immunologists in deciding whether the methods are sufficiently performant for their use case and which of the available models to choose. Additionally, the lack of clearly defined testing standards hinders methodological improvements as the performance of novel methods is difficult to compare. Moreover, the different models are cumbersome to apply from a practical perspective as they employ custom data formats for TCRs and epitopes, discouraging interoperability and limiting their usability for researchers.

We here present ePytope-TCR—an extension to the immune-prediction framework ePytope (formerly FRED2^27^)—to create a simplified interface to TCR-epitope predictors. ePytope-TCR provides a unified framework for 18 general and three categorical pre-trained models provided alongside their original publication, allowing their application to TCR repertoires from six common data formats. Additionally, we guide researchers in their tool selection by utilizing ePytope-TCR for a thorough benchmark of these pre-trained models on two challenging datasets, focusing on repertoire annotation of single-cell studies,^28^^,^^29^ and predicting cross-reactivity toward epitope mutations.^30^

## Results

### ePytope-TCR provides an extendable interface for TCR-epitope prediction

**Figure 1:**
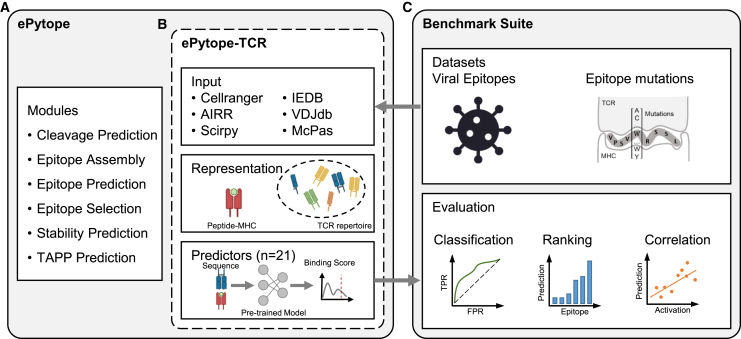
Overview of ePytope-TCR and the benchmark suite (A and B) The existing framework of ePytope (A) was extended for TCR-epitope prediction (B). TCR repertoires can be loaded from six common data formats, and their binding capabilities for pMHCs estimated with 18 general and three categorical sequence-based predictors. (C) The benchmark suite applies ePytope-TCR to two challenging datasets and evaluates the predictive performance for classification, ranking, and correlation.

ePytope (formerly FRED2^27^) represents a framework for T cell epitope detection and vaccine design. Its previous implementation (Figure 1A) covered several immunological steps starting from cleavage prediction to MHC-peptide binding prediction, as well as epitope selection and assembly for vaccination. Several external tools can be conveniently combined through interfaces to standardized data representations of transcripts, variants, proteins, peptides, and MHC alleles.

To enable the binding prediction between epitopes and TCR repertoires (Figure 1B), we extended ePytope with two data structures. Here, adaptive immune receptors consist of one or several immune receptor chains with known CDR3 amino acid sequences and optional V-, D-, and J-genes in addition to metadata such as T cell type and species. Epitopes represent the combination of peptide sequence and, optionally, its binding MHC allele through the aforementioned data representations, enabling full interoperability with the remaining framework. To allow users to incorporate their repertoires, ePytope-TCR provides functionality to load TCRs from common single-cell and bulk formats such as the AIRR standard,^31^ the cellranger-vdj output, and scirpy data object.^32^ Additionally, TCRs can be loaded in the formats of the IEDB,^13^ VDJdb,^14^ and McPAS-TCR,^15^ which represent the three most common public databases of TCR-epitope pairs.

Based on the input of AIR repertoires and epitopes, specificity predictors can be used to estimate binding between all individual combinations of TCRs and pMHCs. While we support 18 general and three categorical predictors for this work (detailed overview in Table 1), novel predictors can be easily integrated by following the defined interface. We utilized the flexibility of ePytope-TCR to evaluate the predictors on two datasets in a standardized fashion via the benchmarking suite (Figure 1C). Overall, ePytope-TCR offers an extendable and easy-to-use tool for applied researchers to predict binding specificity between TCRs and epitope-MHCs. At the same time, it can be used to evaluate new models and make them accessible to the community.

**Table 1:** Overview of TCR-specificity predictors

Name	Date	Training data	# Samples	Pretraining	CDR3	V-, J-genes	Representation	MHC	Network type	Negative pairs	General
ATM-TCR^33^	07/2022	IEDB, VDJdb, McPas	128,142∗	**✗**	β	**✗**	embedding	**✗**	attention	internal (1:1)	**✓**
AttnTAP^34^	08/2022	VDJdb∗	38,134∗	**✗**	β	**✗**	embedding	**✗**	RNN, MLP, attention	internal (1:1)∗	**✓**
BERTrand^35^	06/2023	VDJdb, McPas, Others	32,523	**✓**	β	**✗**	embedding	**✗**	attention	external (1:3)	**✓**
DLpTCR^36^	08/2021	VDJdb, Others	5,710∗	**✗**	β∗	**✗**	1-hot+PCP	**✗**	MLP,CNN	external (1:1)	**✓**
epiTCR^37^	04/2023	IEDB, VDJdb, McPas, Others	106,576∗	**✗**	β	**✗**	PCP	**✗∗**	RF	internal (1:30)	**✓**
ERGO^19^	07/2019	VDJdb∗	35,201∗	**✓∗**	β	**✗**	1-hot∗	**✗**	MLP∗	internal (1:5)	**✓**
ERGO-II^1^	04/2021	VDJdb∗	34,886∗	**✗∗**	β(,α)	(**✓)**	1-hot∗	(**✓)**	MLP∗	internal (1:5)	**✓**
ImRex^20^	12/2020	VDJdb	14,188∗	**✗**	β	**✗**	PCP	**✗**	CNN	internal (1:1)	**✓**
iTCep^38^	05/2023	IEDB, VDJdb, McPas	10,759	**✗**	β	**✗**	PCP	**✗**	CNN	external (1:1)	**✓**
MixTCRpred^18^	09/2023	IEDB, VDJdb, McPas, Others	17,715	**✗**	α,β		embedding	**✗**	attention	both (1:5)	**✗**
NetTCR-2.2^39^	10/2023	IEDB, VDJdb	9,065	**✗**	α,β		PCP	**✗**	CNN	internal (1:5)	**✓**
NetTCR-Cat^39^	10/2023	IEDB, VDJdb	9,065	**✗**	α,β		PCP	**✗**	CNN	internal (1:5)	**✗**
PanPep^40^	03/2023	IEDB, VDJdb, McPas, Others	32,080∗	**✗**	β	**✗**	PCP	**✗**	attention	internal (1:1)	**✓**
pMTnet^41^	09/2021	IEDB, VDJdb, McPas, Others	32,607	**✓**	β	**✗**	PCP	**✓**	CNN,LSTM	internal (1:10)	**✓**
STAPLER^42^	04/2023	IEDB, VDJdb, McPAS, Others	3,924	**✓**			embedding	**✗**	attention	internal (1:5)	**✓**
TCellMatch^21^	08/2019	IEDB∗	9,697∗	**✗**	β∗	**✗**	PCP∗	**✗**	MLP∗	internal (1:1)	**✓**
TCRGP^16^	02/2019	VDJdb, Others	14,469	**✗**	β∗	**✗∗**	PCP	**✗**	GP	internal (1:1)	**✗**
TEIM^43^	03/2023	VDJdb, McPas, Others	45,481	**✗**	β	**✗**	embedding	**✗**	CNN	internal (1:5)	**✓**
TEINet^44^	10/2022	VDJdb, McPas, Others	44,682∗	**✓**	β	**✗**	embedding	**✗**	CNN, attention	internal (1:1)∗	**✓**
TITAN^45^	04/2021	VDJdb, Others	23,145	**✗**	β		SMILES, PCP	**✗**	attention	internal (1:1)	**✓**
TULIP-TCR^46^	07/2023	IEDB, VDJdb, McPas	209,779	**✗**	α,β	**✗**	embedding	**✓**	attention	**✗**	**✓**

The predictors supported by ePytope-TCR are listed with date of publication, model, and training characteristics. The first occurrence in a journal, conference, or preprint is considered the publication date. The table indicates the characteristics of the model version with the highest area under the curve (AUC) in the viral benchmark. If alternative options were additionally available or presented in the publication, the corresponding entry is marked with a star. Optional model inputs are listed in round brackets. # Samples refers to the number of positive training samples. Representation can be either physicochemical properties (PCP), one-hot encoding (1-hot), learned embeddings (embedding), or SMILES representation (SMILES).^47^ Network type refers to the backbone architecture of the predictor without the classification head, consisting of multilayer perceptron (MLP), convolutional neural networks (CNN), recurrent neural networks (RNNs) such as LSTMs or GRUs, and attention- or transformer-based networks (attention). Negative data for training is formed either using the sequences from the matching TCR-pMHC pairs (internal) or by using an additional reference set (external) at a positive-negative ratio of (1:n). General indicates whether the epitope is treated as a model input (**✓**) or as a category (**✗**). See also Table S1.

### Predictors

In an extensive literature review (STAR Methods, Tool selection), we collected a corpus of 18 sequence-based TCR-epitope specificity predictors (Tables 1 and S1). As a prerequisite for integration into ePytope-TCR, the pre-trained models must have been publicly available, and the models were required to predict arbitrary 9-mer epitopes to allow researchers to apply the method to their own TCR repertoires without retraining the model. As a comparative baseline, we further selected three categorical models comprising one of the first predictors and two recent methods indicating high performance.

The resulting methods mainly differ in training data, input information, and network style. All models were trained on combinations of the three major databases IEDB,^13^ VDJdb,^14^ and McPAS-TCR,^15^ in addition to smaller datasets^48^ or sequencing studies.^41^,^49^ However, this prohibits fairly evaluating pre-trained models of the methods on these publicly available databases pairs to avoid leakage between test and training data. While all tools required the CDR3β amino acid sequence as input, several methods also used the CDR3α sequence, the full TCR sequences, CDR1 and CDR2, or MHC type as well. As two exceptional cases, DLpTCR^36^ additionally offers a separate CDR3α-alone model, and ERGO-II^1^ takes the α-chain sequence and trainable embeddings for categorical V- and J-gene information, MHC type, and cell type as optional input. Based on the used databases, the publication date, and the required filtering, the amount of used training data varied drastically between 3,924 and 209,799 positive TCR-epitope pairs. Experimentally validated non-binders are typically not captured in public databases. However, all methods except TULIP-TCR explicitly require negatively annotated pairs during the model training, which is a crucial design choice of the developers as shown by Dens et al.^23^ Negative pairs were either created by randomly shuffling the TCR-epitope combinations from the positive pairs (internal), by matching epitopes to repertoires of TCRs with unknown specificity (external), or both at ratios from 1:1 to 1:30 between the positive pairs from the databases and these negative combinations.

The majority of models encoded the amino acid sequence either via trained embedding layers or through different physicochemical properties (PCP), while one-hot encodings or other representations were less common. Most of the deep learning models employed a multilayer perceptron (MLP) to classify binding vs. non-binding, which was often preceded by a feature-extracting network. Here, all common network styles and combinations of them have been tested with attention-based neural networks and convolutional neural networks (CNNs) outnumbering recurrent neural networks (RNNs). As two exceptions, epiTCR employed random forests (RFs) and TCRGP a Gaussian process (GP) instead of deep neural networks. Interestingly, TULIP-TCR was not directly trained for classification but in an unsupervised fashion to learn the implicit dependencies among TCR, epitope, and MHC, thereby completely avoiding the need for generating negative training pairs. In a similar direction, several models employed autoencoding (AE)- or masked language model (MLM)-styled pretraining to learn a TCR representation from a large corpus of TCRs with unknown specificity.

### Strong biases prevail between different viral targets

**Figure 2:**
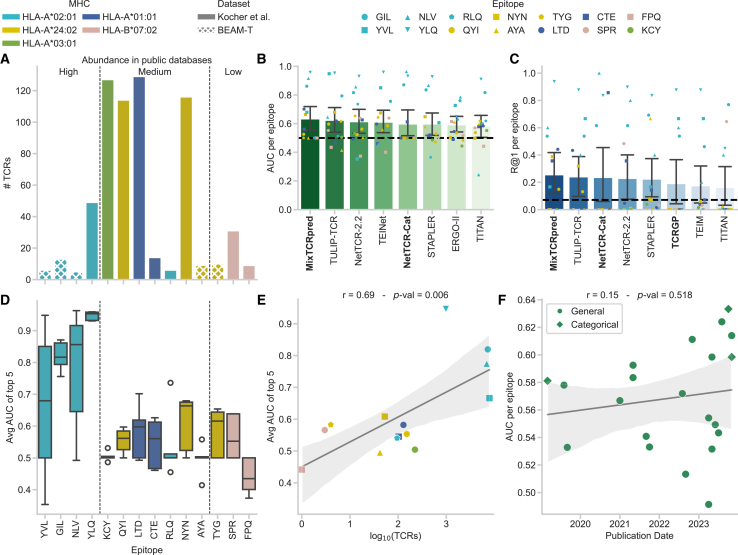
Benchmark on viral epitopes (A–C) (A) The dataset contains 638 TCR-epitope pairs stemming from 14 epitopes, five MHC types, and four diseases. The epitopes are ordered based on the amount of matching CDR3β sequences in public databases in high (*n* ≥ 500), medium (*n* ≥ 4), and low (*n* < 4) abundance. The best eight predictors measured by the area under the curve (AUC) (B) and recall at 1 (R@1) (C) calculated per epitope. Categorical models are marked with boldface names. The mean indicates the average metric score measured for each epitope individually, while the error bars indicate the 95% confidence interval over the scores (*n =* 14 epitopes). The dashed black line marks random predictions. (D) AUC scores per epitope of the five best-performing predictors (*n =* 5 models). The boxplot indicates the data quartiles, while the median is indicated as a horizontal line. Outliers are marked separately. (E) Pearson correlation between the average AUC score of the predictors from (D) and the amount of unique CDR3β-epitope pairs available in the combined databases IEDB,^13^ VDJdb,^14^ and McPas-TCR^15^ over all epitopes (*n = 14*). (F) Pearson correlation between the AUC scores averaged over the epitopes and the initial publication date of the method. The line represents the mean of the data with the 95% confidence interval as an error band. See also Figures S1–S6 and Tables S2 and S3.

We investigated to what degree TCR-epitope predictors can annotate repertoires from sequencing studies. Therefore, we simulated the annotation of a single-cell dataset of 638 TCRs specific to viral epitopes with all 21 predictors through ePytope-TCR. This repertoire was obtained by combining the severe acute respiratory syndrome coronavirus 2 (SARS-CoV-2) vaccine study from Kocher et al.^28^ (*n =* 595 TCRs, m = 14 epitopes) with the sample datasets of the BEAM-T pipeline of 10x Genomics^29^ (*n =* 43 TCRs, m = 5 epitopes). As a ground truth, we utilized the specificity assigned via multimer staining to one of 14 epitopes from influenza A virus (IAV), Epstein-Barr virus (EBV), cytomegalovirus (CMV), and SARS-CoV-2 bound to five different MHC alleles. The original study^28^ conducted strict quality control and validation, surpassing most single-cell specificity assignments, which limits the amount of false annotation in this evaluation data (see STAR Methods). As expected in repertoires from sequencing samples, the distribution of TCRs binding to the different epitopes is skewed (Figure 2A) as the four most represented epitopes bind to 76.2% of the TCRs (*n =* 486) while five epitopes are represented fewer than 10 times. We predicted the binding score between each TCR-epitope pair while considering the assigned epitope for each TCR as positive samples. For classification metrics, we additionally required negative pairs during this evaluation, for which we used the 13 other epitopes for each TCR. While this resembles internal shuffling as used by most methods during their training phase (Table 1), none of these negative TCR-epitope pairs were detected as positive binders in the underlying sequencing studies and are, therefore, more likely to be true negatives.

In the first step, we evaluated the performance based on area under the curve (AUC) as a common metric for general classification and TCR-epitope prediction. The metric was calculated across the dataset and for each epitope separately (Figures 2B and S1A; Table S2). Only four general and two categorical methods achieved an average AUC greater than 0.6, with MixTCRpred performing best at a score of 0.63 ± 0.17 (*n =* 14 epitopes). Eight methods were less than 5% points off the threshold of 0.5. While this average at first glance resembles the random prediction baseline, all eight models achieved an AUC value greater than 0.7 on at least one epitope for which more than 40 CDR3β sequences are currently contained in public databases (Table S3). Overall, the best-performing predictors remained similar across other classification metrics with a maximal average precision score (APS) of 0.25 ± 0.28 and an F1 score of 0.23 ± 0.26 (Table S2). When a method provided multiple models due to cross-validation or testing different design choices, we reported the version with the highest AUC in this test and kept this version for the remainder of the benchmark. As this selection slightly favors methods reporting multiple models, the achieved performance should be considered an optimistic estimate for each model family, especially for cross-validated methods. Regarding design choices, the most prominent difference between models of the same method was the data filtering strategies and the training dataset, with models trained on the VDJdb typically outperforming models trained on McPAS-TCR (Figure S1B).

In practice, the models do not directly classify a TCR-epitope pair as binding or non-binding but provide a continuous prediction score with high values indicating a high binding probability. To assign specificity, practitioners would be required to define thresholds on this prediction score to decide whether a T cell is specifically binding to an epitope. However, suggestions for binding score thresholds are often not provided in the original publication. Rank-based metrics offer a more intuitive evaluation in this setting (Figures S1C and S1D; Table S2) as thresholds are difficult to estimate as the score profile between binders and non-binders overlaps strongly (Figures S2A–S2U). For each TCR, the prediction scores for the 14 epitopes were ordered, and the position (average rank) of the correct match was evaluated with 7.5 as the middle between 1 and 14, indicating random prediction. The recall at K (R@K) indicates how often the correct epitope occurred within the K highest scores (random for R@1: 1/14 = 0.071). The three methods with the highest score—MixTCRpred, TULIP-TCR, and NetTCR-Cat—achieved an R@1 between 0.24 and 0.26 and R@3 between 0.38 and 0.40 (Figure 2C). While these predictors exceed the random prediction threshold of 0.071, applying them for annotation would lead to correct annotation of TCRs in only one-fourth of cases, which is insufficient for downstream analysis.

These shortcomings were caused by large discrepancies in performance between the different epitopes, as indicated by a large standard deviation in R@1 greater than 0.25 and in AUC greater than 0.15 of the best five predictors (Figures 2B and 2C; Table S2). For five epitopes, at least one predictor reached an AUC greater than 0.75 with a maximum of 0.98 for TEIM on the YLQPRTFLL epitope (Table S3). Notably, four of these five epitopes were reported with 500 or more matching CDR3β sequences (high abundance) in public databases and were bound by *HLA-A∗02:01* (Figure 2A), one of the most extensively studied human HLA alleles (Figure 2D). However, for five epitopes, no predictor reached an AUC greater than 0.7. These comprised all previously almost unobserved epitopes occurring in public databases with three or fewer TCRs (low abundance), but also two out of seven epitopes with an abundance of four to 500 CDR3β sequences (medium abundance). Naturally, the three categorical methods did not provide any model for the almost unobserved epitopes but demonstrated higher performance than general models on four epitopes from other classes, indicating that general models fail to utilize synergistic effects across epitopes. While MixTCRpred provided models for more epitopes and, thereby, achieved the highest average score, the categorical version of NetTCR-2.2 performed slightly better on most epitopes with available models (Table S3).

As shown previously,^18^^,^^26^^,^^50^ differences in performance between epitope classes were caused by the over-representation of some epitopes in public databases as indicated by a strong significant Pearson correlation of *r =* 0.69 between the average AUC score of the five best predictors and the logarithmized amount of CDR3β-sequences in public databases per epitope (Figure 2E). Interestingly, the average AUC score correlated even stronger (*r =* 0.74) with the logarithmized amount of αβ-paired CDR3 sequences (Figures S3A and S3B), as four out of the five best-performing methods utilized both TCR chains in their prediction. While the availability of paired sequences was statistically significantly related to the number of CDR3β sequences (*r =* 0.79), RLQSLQTYV and KCYGVSPTK form two notable exceptions (Figure S1C). Despite 94 and 223 observed CDR3β sequences, only six and zero were recorded with corresponding α-sequences, respectively, explaining the low predictive performance on these two epitopes. This bias toward epitopes with a large number of TCRs available for training was also prevalent in the average predictive performance within each group: While for high abundance the best predictor achieved an AUC of 0.85, the scores decreased to 0.57 and 0.59 for medium and low, respectively. Interestingly, ERGO—one of the first published methods—achieved the highest average AUC in the low abundance setting. Presumably, much of the performance increase in recent years (Figure 2F) can be attributed to the rise of publicly available TCR-epitope pairs (Figures S4A and S4B), especially through SARS-CoV-2 epitopes forming the majority of this dataset, while choices in method design had less influence on the performance (Figure S4C). While these performance differences between epitopes clearly hinder the applicability of predictors toward less-observed epitopes, this shortcoming may often be overlooked when calculating metrics across the whole dataset or weighting them by epitope frequency, which will bias the evaluation toward highly studied epitopes.

Besides large differences in performance, we additionally observed strong biases in the prediction scores between the epitopes. For 14 of 21 methods, we observed a decrease in performance when the AUC was calculated for all epitopes simultaneously compared with the averaged AUC calculated for each epitope individually (Figure S5). This indicates that the predictors were better at ordering TCRs within epitopes, but the prediction scores of the models are not comparable between the different epitope targets. This becomes even more apparent when investigating the average prediction score per epitope across the whole dataset (Figure S6A). An ideal predictor would assign a score of 1 to all correct binders and 0 otherwise and, thereby, match the epitope frequencies of the dataset. However, 12 predictors have mean binding scores greater than 0.50 for at least one epitope compared with a maximal epitope frequency of 0.20. This indicates that predictors overestimated binding to certain epitope classes, while presumably less-observed epitopes are always predicted as non-binders. This is further displayed by the fact that some predictors show low standard deviation in the prediction scores of all TCRs against an individual epitope, with an average smaller than 0.10 for four predictors (Figure S6B). This indicates that these predictors always provide highly similar prediction scores for a given epitope, regardless of the TCR sequence. Presumably, epitopes frequent in the training data were always classified as binding, while others were predicted as non-binding. TULIP-TCR could not be considered in this analysis, as the binding score is not scaled between 0 and 1. However, the remaining best-performing methods in AUC and R@1 except MixTCRpred all had an overall mean prediction score below 0.2, leading to a profile resembling the true frequencies (Figure S6A). Such a comparison between epitopes may be overlooked when evaluating metrics only per epitope. However, prediction scores are required to be comparable across epitopes when annotating repertoires, as otherwise, epitope-specific thresholds need to be defined. Potentially, these shortcomings could be avoided by normalizing against the prediction score of suitable background TCRs, which are presumably unspecific to the epitope in question and can thereby be used to estimate the prediction score level.

In summary, our findings suggest that current TCR-epitope prediction tools struggle to accurately assign specificity to viral epitopes in TCR sequencing repertoires in a global manner. While several methods show strong performance for commonly observed epitopes in public databases, their generalizability to less-represented or unobserved epitopes is limited, with prediction results resembling random chance. Categorical methods often performed on par or better compared with general methods, if a pre-trained model was provided for an epitope, indicating that general methods failed to utilize synergetic effects across different targets. Additionally, significant biases exist between the prediction scores of different epitopes, complicating their comparability between targets. As a result, we recommend using these predictors primarily for epitopes that are well-represented in the common databases and defining distinct thresholds for each target.

### Epitope mutations are challenging for general specificity predictors

**Figure 3:**
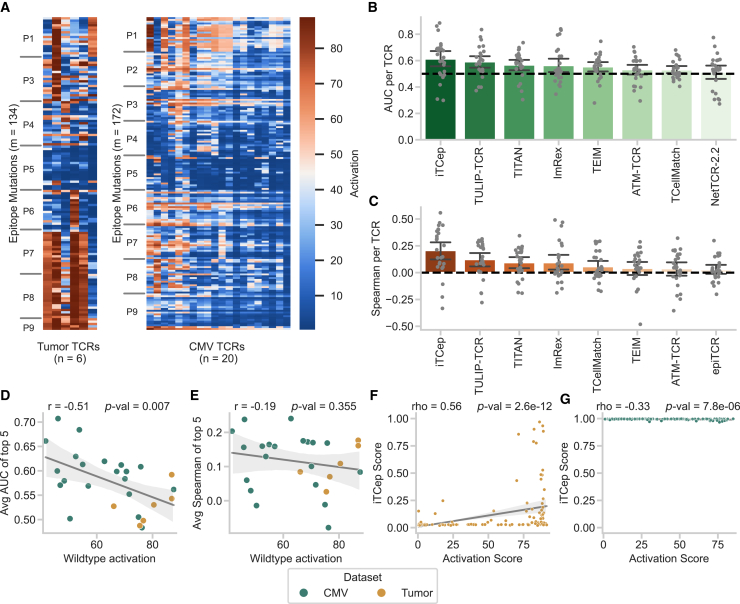
Benchmark on TCR reactivity against epitope mutations (A–C) (A) The dataset consists of two deep mutational scans of TCRs reactive to mutations of the neo-epitope VPSVWRSSL and the human CMV epitope NLVPMVATV. Prediction performance for this dataset is shown for the best eight predictors, measured by the area under the curve (AUC) (B) and Spearman correlation coefficient (Spearman) (C) calculated per TCR. The mean indicates the average metric score measured for each TCR individually, while the error bars indicate the 95% confidence interval over the scores (*n =* 26 TCRs). (D–G) Pearson correlation (*n =* 26 TCRs) between the average AUC (D) or Spearman coefficient (E) and the activation score to the wild-type epitope. Each point represents one TCR with the performance scores averaged for the best predictors (*n =* 5) on the corresponding metric from (B) and (C), respectively. Spearman correlation between the prediction score of iTCep and the activation score for all epitope mutations for two selected TCRs with the highest (F, *n =* 133 epitopes) and the lowest (G, *n =* 172 epitopes) correlation score. The line represents the mean of the data with the 95% confidence interval as an error band. See also Figures S7–12 and Tables S4–6.

As a second test, we utilized two deep mutational epitope scans from our previous study^30^ generated via high-confidence, low-throughput experiments. This dataset comprised continuous activation scores of six TCRs against 132 single amino acid mutations of the neo-epitope VPSVWRSSL, and 172 mutations of the human CMV epitope NLVPMVATV (Figure 3A). All selected TCRs recognized the corresponding wild-type epitope with high functional avidity but vary in reactivity toward their mutations. Predictions on mutational effects could depict potential immune escape of pathogens or identify cross-reactive TCR candidates for cancer immunotherapies. However, estimating reactivity against epitope mutants represents one of the most challenging tasks for TCR-epitope predictors, as the model must be highly sensitive toward single amino acid changes in the epitope input. Again, we predicted binding with ePytope-TCR for all epitope-TCR combinations and evaluated the AUC calculated per TCR (Figure S7A; Table S4) based on the activation score threshold of the original publication^30^ to define activating and non-activating TCR-epitope pairs. As the categorical models did not provide a model for the epitope mutations, we focused solely on general methods in this test. The three best-performing predictors were iTCep at an AUC of 0.61 ± 0.15 (*n =* 26 TCRs) (Figure 3B), a method that did not achieve top eight performance on the viral dataset (Figures 2B and 2C), TULIP-TCR (0.59 ± 0.11), and TITAN (0.57 ± 0.10).

The data on mutational epitope scans additionally provide a continuous score for each TCR-epitope pair relating to the fraction of T cells from this clonotype that were activated by a mutated epitope peptide. As a next step, we, therefore, evaluated to what degree the models’ prediction scores correlate with the respective activation scores in order to investigate to what degree continuous T cell activation was captured by the predictors. The overall performance largely followed the classification setting (indicated by AUC in Figure 3B) on this dataset and was with a maximum of 0.21 ± 0.22 in Spearman rank coefficient close to the random prediction boundary of 0.0 (Figures 3C and S7B; Table S4). Interestingly, a variant of TCellmatch—one of the first general epitope-TCR predictors—achieved the highest AUC (0.62 ± 0.11) and Spearman coefficient (0.29 ± 0.16) on this dataset (Figures S8A and S8B) but is not listed in the results as it is not the version selected in the previous evaluation (Figure S2). These findings suggested that none of the methods so far is suitable for predicting the effect of point mutations in epitopes.

As in the viral dataset, we observed large differences in performance between the different TCRs and targets. We observed a significant negative Pearson correlation (*r = −*0.51) between the average performance scores of the best five general predictors in AUC (Figure 3B) to the activation score of the TCRs (*n =* 26) against the wild-type epitope (Figure 3D). The correlation (*r = −*0.19) of the best five models (Figure 3C) was not significant for the Spearman coefficient (Figure 3E). Predictive performance for TCRs of both subsets varied greatly. Only iTcep performed within the best five models measured by AUC for the CMV and the neo-epitope, and four methods had an AUC greater than 0.5 (Figures S9A–S9C) or Spearman correlation greater than 0.0 (Figures S9D–S9F) on both subsets (Table S5). Further, we did not observe any correlation of the evaluation metrics between both datasets (Figures S9C and S9F). To validate performance difference between base epitopes, we conducted this test on a deep mutational scan of murine TCRs reactive toward the model epitope SIINFEKL (Figure S10A). We excluded this dataset from our initial benchmark to keep the evaluation set constant, as four of the 18 models could not be applied due to restrictions regarding epitope length and murine MHCs or TCRs. As in the previous test, the overall performance was limited, with STAPLER achieving a maximum AUC score of 0.58 ± 0.16 and TULIP-TCR having the highest Spearman correlation of 0.15 ± 0.16 (Table S6). Interestingly, methods trained on human and murine TCRs surpassed most models developed on only human data (Figures S10B and S10C), even when they showed limited performance in previous tests. This indicates that the models vary strongly between different base epitopes and, therefore, no conclusive performance estimations can be made regarding the predictions for other deep mutational scans.

Again, we observed a strong bias between the different target epitope variants with regard to the continuous prediction scores that the models provide as an output to indicate binding. Even for the two leading methods, iTCep and TULIP-TCR, the prediction scores between both base epitopes separated into a clear bimodal distribution (Figures S11A–S11R). Fourteen of the 18 tested methods yielded lower average prediction scores for the tumor epitope mutations (Figures S12A and S12B) despite them surpassing the activation threshold by 38.9% more often than the CMV epitopes. As NLVPMVATV is one of the epitopes with the most recorded TCRs in public databases, prediction methods were inherently biased to assume binding with a higher probability compared with the unobserved VPSVWRSSL neo-epitope, which is reflected by a higher level of prediction scores. This can be illustrated well in the example of the best-performing method, iTCep. The TCR with the highest Spearman coefficient of 0.56 (R25, tumor) showed variable prediction scores between 0 and 1 (Figure 3F). While many mutations would be incorrectly classified as non-binding based on low prediction scores, several epitopes show higher scores, indicating that the model successfully incorporated the epitope sequence into its prediction. In contrast, all mutations are predicted to bind for the TCR with the lowest coefficient of −0.33 (CMV81-14, CMV) at a prediction score greater than 0.95 with a standard deviation of 0.005 (Figure 3G). Apparently, the model correctly predicted the binding of the TCR toward the base epitope but failed to take the negative effects of mutations into account, resulting in high prediction scores for all mutations. This again shows the biases that different epitope targets imprint on the prediction score based on their prevalence in public databases.

In summary, general TCR-epitope predictors cannot reliably predict the effect of epitope mutations. This highlights the need for specialized datasets and prediction models such as P-TEAM, which was proposed by us recently.^30^ We observe strong biases between different target classes, highlighting the data dependencies of current predictors. Of the best models in the viral dataset, TULIP-TCR is also ranked high in the mutation dataset. While no current model can be utilized for practical use in this setting, we hope that this benchmark sparks research interest, and we expect a large potential for predicting epitope reactivities in future works.

## Discussion

*In silico* binding prediction of a TCR toward pathogen-, tumor-, or self-derived epitopes will signify a pivotal step in computer-aided vaccine and immunotherapy development, as well as for the investigation of TCR repertoire evolution. While several methods have been proposed to predict specificity based on the TCR and epitope sequences, they have not yet found wide application in immunological research. On the one hand, these proposed approaches widely lack interoperability among themselves and with standard repertoire data formats. On the other hand, their predictive performance in applied use cases has remained unknown. Despite notable efforts by the ImmRep workshop,^24^^,^^25^^,^^26^ many pre-trained models of the predictors have not been systematically evaluated under benchmark conditions, and no defined testing rules and datasets exist to guide the development of new methods.

To alleviate these shortcomings, we here introduce ePytope-TCR as a TCR-epitope prediction extension of the vaccine design and immuno-prediction framework ePytope (formerly FRED2^27^). We incorporated a unified interface to 18 general and three categorical previously published pre-trained TCR-specificity predictors and import utilities to three common databases and three standardized repertoire data formats. We showcase the capabilities of ePytope-TCR by applying the predictors to two benchmarking datasets, which test their ability to annotate single-cell datasets with specificity toward viral epitopes and to predict the cross-reactivity of TCRs against epitope mutations.

Despite overall low metric scores for classification and ranking when averaged across targets, we found sufficient performance for epitopes with large support in public databases, with AUC scores greater than 0.75 on five of the tested epitopes. These results are in agreement with previous findings that TCR-epitope prediction is still limited by the lack of diverse training data, leading to failures of predictors on unseen targets.^22^^,^^51^ Evidently, general models did not benefit from synergistic effects between epitopes, as further shown by often lower performance than categorical models for more abundant targets. However, we observed increasing performance in recent models, which may partially be attributed to the rise in available TCR-epitope pairs. Therefore, we advise applying these predictors only for target epitopes covered in the methods’ respective training data.

While the predictors were capable of annotating specificity for selected epitopes, they were not able to reliably predict the effect of mutations in known or unknown wild-type epitopes on T cell function. We hope this benchmark encourages further interest in this use case as one of the most challenging tasks in TCR-epitope prediction.

In both datasets, we observed a strong bias in the prediction scores dependent on the target epitope. This further complicates applying the predictors for annotation as they therefore require epitope-specific classification thresholds. Both the different performance between epitopes and the bias in prediction scores highlight important aspects for the evaluation of TCR-epitope predictors. On the one hand, the metric scores are skewed toward frequently observed epitopes, so low performance on rare epitopes might not be discovered when metrics are evaluated across datasets or weighted by class support. On the other hand, biases in prediction score levels might not be discovered when evaluating only within epitope classes. Therefore, we advise performing both evaluations separately to allow a holistic view of the models’ performance.

Our contribution to TCR-epitope prediction in this work is 2-fold. First, we make TCR-epitope prediction methods available for the applied community by providing an interoperable interface with ePytope-TCR, which allows researchers to apply the predictors to their own repertoire data. Through the benchmark, we further guide them in which settings the models can be applied, which mainly reduces them to settings where the epitopes have been well-researched. Second, we provide the basis for accelerating future method development by defining evaluation datasets and metrics and allowing fast benchmarking to other methods through ePytope-TCR. Ultimately, we believe that enhanced evaluation methods and improved interoperability will bridge the gap, making TCR-epitope predictors more applicable in immunological studies of large-scale TCR repertoires, vaccine development, and the identification of therapeutic TCR candidates for pathogens and tumors.

### Limitations of the study

This evaluation provides an overview of the performance of currently available general TCR-epitope predictors. However, our tests are—similar to the methods themselves—limited by the data at hand. First, we only evaluated predictions for CD8+/MHC-I epitopes as this is the major focus of current research. To enable a complete evaluation between all predictors, we disregard all non-9-mer epitopes, leading to a limited amount of 638 TCRs in the viral dataset and two scans in the mutation dataset. Further, the epitopes were bound by only five different MHC classes, which did not allow us to observe strong biases between alleles independent of the number of reported TCRs. Additionally, most epitopes of the viral dataset were observed in public databases at least once. An improved benchmark would ideally contain several epitopes without any reported binding TCRs to evaluate generalization for out-of-target predictions. Despite our best efforts, we also cannot rule out a small amount of wrongly annotated TCRs in the viral benchmark, as multimer staining and the resulting annotation may be susceptible to background noise. Finally, in this work, we evaluated the performance of pre-trained models and did not retrain any model ourselves. Therefore, we cannot separate whether differences in performance were caused by the methods’ design choices or by their underlying training data. A standardized test of different architectures, training paradigms, data filtering, TCR information, and pretraining strategies remains open for future research.

## Resource availability

### Lead contact

Further information and requests for resources should be directed to and will be fulfilled by the lead contact, Benjamin Schubert (benjamin.schubert@helmholtz-munich.de).

### Materials availability

This study did not generate new unique reagents.

### Data and code availability

• All datasets used in this manuscript have been deposited at Zenodo: https://doi.org/10.5281/zenodo.15025579 and are publicly available and provided through the benchmarking suite. The DOI is listed in the key resources table. The unprocessed data of the SARS-CoV-2 study can be accessed via Zenodo: https://doi.org/10.5281/zenodo.15691612. The raw sequencing data of four samples from 10x Genomics can be obtained on the company’s homepage (S1, S2, S3, S4). The unprocessed data from the mutation dataset can be accessed in the supplementary material of the original publication.^30^
• The software package ePytope-TCR, including tutorials, has been deposited at Zenodo: https://doi.org/10.5281/zenodo.15497114 and is available at https://github.com/SchubertLab/epytope. The benchmarking suite, including the source code to reproduce the results and figures of the evaluation from Data S1–S6, has been deposited at Zenodo: https://doi.org/10.5281/zenodo.15497114 and can be accessed at https://github.com/SchubertLab/benchmark_TCRprediction. The DOIs are listed in the key resources table. The repositories of the individual predictors are linked in Table S1.
• Any additional information required to reanalyze the data reported in this paper is available from the lead contact upon request.

## Acknowledgments

This work was supported by the BMBF grant DeepTCR (#031L0290A) and the de.NBI Cloud within the German Network for Bioinformatics Infrastructure (de.NBI) and ELIXIR-DE (10.13039/501100003163Forschungszentrum Jülich and W-de.NBI-001, W-de.NBI-004, W-de.NBI-008, W-de.NBI-010, W-de.NBI-013, W-de.NBI-014, W-de.NBI-016, and W-de.NBI-022). F.D. is supported by the Helmholtz Association under the joint research school “Munich School for Data Science - MUDS” and acknowledges financial support from the 10.13039/100008662Joachim Herz Stiftung.

## Author contributions

F.D. and B.S. conceived the project and supervised the research. F.D., A.C., and M.A. integrated the models. F.D. and A.C. performed the literature review. F.D. supervised the integration and implemented and performed the evaluation. K.K. and K.S. generated the viral test data and provided it prior to publication. K.K. and K.S. provided feedback on the evaluation and the manuscript. All authors wrote the manuscript.

## Declaration of interests

The authors declare no competing interests.

## STAR★Methods

### Key resources table

**Table:** 

REAGENT or RESOURCE	SOURCE	IDENTIFIER
**Deposited data**		

Generated Datasets	In house	Zenodo: https://doi.org/10.5281/zenodo.15025579

**Software and algorithms**		

Python	Conda	python = 3.8
Numpy	Conda	numpy = 1.20.2
Pandas	Conda	pandas = 1.2.5
SciPy	Conda	scipy = 1.6.2
Custom Code	In house	Zenodo: https://doi.org/10.5281/zenodo.15075019
ATM-TCR	GitHub	https://github.com/Lee-CBG/ATM-TCR
AttnTAP	GitHub	https://github.com/Bioinformatics7181/AttnTAP
BERTrand	GitHub	https://github.com/SFGLab/bertrand
DLpTCR	GitHub	https://github.com/JiangBioLab/DLpTCR
epiTCR	GitHub	https://github.com/ddiem-ri-4D/epiTCR
ERGO	GitHub	https://github.com/louzounlab/ERGO
ERGO-II	GitHub	https://github.com/IdoSpringer/ERGO-II
ImRex	GitHub	https://github.com/pmoris/ImRex
iTCep	GitHub	https://github.com/kbvstmd/iTCep
MixTCRpred	GitHub	https://github.com/GfellerLab/MixTCRpred
NetTCR-2.2	GitHub	https://github.com/mnielLab/NetTCR-2.2
NetTCR-Cat	GitHub	https://github.com/mnielLab/NetTCR-2.2
PanPep	GitHub	https://github.com/bm2-lab/PanPep
pMTnet	GitHub	https://github.com/tianshilu/pMTnet
STAPLER	GitHub	https://github.com/NKI-AI/STAPLER
TCellMatch	GitHub	https://github.com/theislab/tcellmatch
TCRGP	GitHub	https://github.com/emmijokinen/TCRGP
TEIM	GitHub	https://github.com/pengxingang/TEIM
TEINet	GitHub	https://github.com/jiangdada1221/TEINet
TITAN	GitHub	https://github.com/PaccMann/TITAN
TULIP-TCR	GitHub	https://github.com/barthelemymp/TULIP-TCR

### Method details

#### Tool selection

We conducted a literature review by examining ERGO,^19^ NetTCR,^17^ TCellmatch,^21^ and TITAN^45^ as examples of the first generation of general TCR-epitope predictors. Next, we reviewed the references of each publication to identify prior work on this topic. Based on this initial list, we included all prediction-related publications that referenced any of these methods through an iterative process. After composing this comprehensive list in November 2023, we excluded models that did not meet the requirements of this benchmark. Specifically, we removed predictors that treated the epitope as a category or performed atlas-query mapping, as these approaches are not suited for general TCR-epitope prediction. All models were required to make predictions at the sequence level without relying on structural information or modeling. Additionally, the source code and pre-trained model weights had to be released under a license open for academic use. Further, we selected three categorical models as baselines, which represent one of the earliest TCR-epitope predictors and two more recent methods.

#### Predictors

If not stated otherwise, the predictors were integrated into ePytope-TCR based on their original implementation as described in their code repository (Table S1). However, several predictors required minor adjustments listed in the following for reproducibility.

##### pMTnet

In contrast to the other models, pMTnet is trained to indicate a high binding probability by a low score instead of a high score. We, therefore, subtracted the resulting score from one to keep the score direction uniform within ePytope-TCR, with high scores indicating a high binding probability.

##### MixTCRpred

For a robust prediction score across epitopes, we utilized the percent rank as described in the original publication. Similarly to pMTnet, we subtracted its score from one to correct its direction.

##### ERGO-II

To execute the predictor, the original implementation was slightly modified by ePytope-TCR. The model was changed to automatically select the available execution device, and the path to the pre-trained models was changed to point to the correct directory.

##### TULIP-TCR

Similar to ERGO-II, the execution device was adjusted, and unspecified program arguments were exchanged. Additionally, the quantitative score and the used MHC were appended to the output of the predictor.

##### STAPLER

STAPLER requires full TCR sequence information. ePytope-TCR, however, provides the CDR3 sequence in addition to the categorical label of V-, D-, and J-gene, as this is the format provided by most databases. Therefore, Stitchr^52^ was used to recreate the full TCR sequence.

##### NetTCR2.2

The full TCR sequence was obtained as described for STAPLER. Following, the CDR1 and CDR2 sequences for α- and β-chain were identified with ANARCI^53^ as described in the publication.^39^

##### DLpTCR

The α-β version of DLpTCR was excluded from the benchmark as it does not provide a continuous prediction score, but rather a binary label, which is not suitable for the performance metrics chosen in this benchmark.

#### Datasets

The two datasets utilized in this benchmark were publicly available but not yet incorporated into public databases, which was important to avoid data leakage to the models’ training data. The initial data were preprocessed by the benchmarking suite to facilitate a standardized evaluation. Generally, we eliminate TCRs with unknown specificity and missing annotation of CDR3 sequences, V-, or J-genes on α- and β-chain. To maintain consistency in the evaluation set, TCRs with CDR3 length greater than 19 were excluded, along with non-9-mer epitopes, as some predictors are restricted to 9-mer epitopes.

##### Viral datasets

We combined two single-cell datasets stemming from a Severe Acute Respiratory Syndrome Coronavirus 2 (SARS-CoV-2) vaccine cohort study by Kocher et al.,^28^ and the BEAM-T pipeline example datasets provided by 10x Genomics on their company’s website. Both datasets include TCR sequencing and staining with DNA-barcoded peptide-MHC multimers to assign epitope specificity. The vaccine cohort contains TCRs reactive to SARS-CoV-2 epitopes as well as other viral controls to Human Herpes Virus 1 (HHV-1), Influenza A Virus (IAV), and Epstein-Barr virus (EBV) from 14 donors, including a hypervaccinated individual, who at that point has received 215 separate vaccinations.^54^ Peripheral blood mononuclear cells (PBMCs) were sequenced for transcriptome, TCRs, and partially surface protein markers. The specificity was identified through absolute and relative thresholds on UMI counts of peptide-loaded MHC-dextramers separate for each epitope and sample. This annotation was further validated through TCRdist-based sequence similarity within the antigen-specific repertoires.^7^ Additionally, 106 clones were re-expressed in primary human cells and tested for their specificity with a hit rate of 92%. The underlying annotated data object can be obtained from Zenodo, from which we collected clonotypes for all 9-mer epitopes described in the publication. For the BEAM-T data,^29^ the raw sequencing data for four samples were downloaded from the 10x Genomics website (accessed April 2024), which contain single-cell transcriptome, TCR, and multimer-staining against eleven viral epitopes from human Cytomegalovirus (CMV), EBV, IAV, and SARS-CoV-2. The raw data were processed using the ’cellranger multi’ command with cellranger (7.1.0). All cells without identified TCRs were removed, and specificity was assigned based on the BEAM algorithm’s score at a threshold of 92.5% as suggested by 10x Genomics. Cross-reactive TCRs based on this definition, either on a cell- or on a clonotype-level, were removed from the dataset. Non-9-mer epitopes and epitopes bound by fewer than five TCRs in the joint dataset were removed. Additionally, we removed 48 TCR-epitope pairs due to overlaps with the IEDB,^13^ VDJdb,^14^ or McPAS-TCR,^15^ which further increases confidence in the accuracy of the annotation. Finally, the combined viral dataset resulted in a total of 638 TCRs, which were reactive to one of 14 epitopes bound to one of five different MHC alleles. Overall, the quality control and validation conducted in the original study surpass the acceptance criteria of public databases and most single-cell analyses. While a small portion of TCRs might still be falsely annotated, the impact of the underlying benchmark test is likely minimal and cannot be avoided in any form of evaluation.

##### Mutation dataset

This dataset originated from our previous publication,^30^ in which we studied and predicted the effect of epitope mutations on T cell activation. The dataset comprises three TCR repertoires with annotated activation scores toward deep mutational epitope scans, i.e., each epitope residue was systematically exchanged by all 19 other amino acids. Details on data acquisition can be found in the original publications.^30^^,^^55^ In short, for each repertoire TCRs with high avidity toward the wildtype epitope were expressed in Jurkat triple parameter reporter cells (JTPRs) and co-incubated with peptide-pulsed splenocytes. For each combination of mutated epitopes and TCRs, a continuous activation score was assessed through NFAT reporter expression in flow cytometry. While such low-throughput experiments are limited to a small number of TCRs tested simultaneously, they allow the quantification of such functional avidity with high confidence. Many predictors were predominantly trained on human TCRs and MHCs, and both DLpTCR and STAPLER accept only 9-mer epitopes as input. Therefore, we selected the two human datasets, which contain TCRs against the neo-epitope VPSVWRSSL and the human CMV epitope NLVPMVATV. Following the original publication, the activation score was binarized by the thresholds of 66.09% and 40.0% for the neo-antigen and CMV datasets, respectively, representing active T cell recruitment and activation. TCR-pMHC pairs of the TCR R27 were excluded from the neo-antigen dataset as no peptide surpassed the binarization threshold. The corresponding HLA alleles *HLA-B∗07:02* for the neo-epitope and *HLA-A∗02:01* for the CMV epitope were added as an annotation to each TCR-epitope pair. As all peptides stem from single point mutations of the 9-mer epitopes and the TCR CDR3 sequences lie within the allowed ranges of all predictors, no additional TCR-pMHC pairs were filtered.

Over time, these datasets may be added to public databases of TCR-epitope pairs. For the viral dataset, we made sure that no CDR3-epitope pair was present in the database during the development of the predictors. Although some TCR-wildtype epitope pairs from the mutation dataset may have been part of a training dataset for the selected prediction model, none of the CDR3 and mutated epitope pairs are included in any of the commonly used TCR-specificity databases. We propose that other methods evaluated with the benchmarking suite carefully filter their training and validation sets for TCR-epitope pairs following the rules outlined here.

#### Metrics

All predictors provide a continuous score to indicate the binding of a TCR to an epitope, where higher scores imply a higher chance of binding. To evaluate the models, we employed classification metrics (AUC, APS, F1-Score), rank-based metrics (Recall at K [R@K], Average Rank), and correlation-based metrics (Pearson correlation, Spearman rank coefficient). For the classification metrics, a binding TCR-epitope pair was considered as a positive sample. For the viral dataset, all other possible combinations of TCR-epitope were considered negative samples. All samples not exceeding the binarization thresholds in the mutation dataset were considered non-binding. If not indicated otherwise, we refer to the metrics calculated within groups, i.e., per epitope in the viral dataset and per TCR in the mutation dataset. Additionally, we report the metrics macro-averaged across the whole dataset, i.e., each class in the group has equal weight.

##### AUC, APS

The Area Under the Receiver Operating Characteristic (AUC) and Average Precision Score (APS) both summarize the prediction quality across all classification thresholds, indicating how well the predicted value separates positive from negative pairs. While the AUC calculates the area beneath the curve of Recall vs. False positive rate, the APS utilizes the Precision-Recall curve.

##### F1-score

The F1-Score represents the harmonic mean between recall and precision. To determine the classification boundary, the F1-score on all prediction values was evaluated individually for each predictor and dataset, and the threshold resulting in the highest value was chosen.

##### R@K

Given a repertoire of TCRs that are known to bind to a limited set of epitopes, rank-based metrics describe how well the correct epitope can be identified for each TCR. Following its definition as described by Oh Song et al.,^56^ we consider the R@K as the average of how often the correct epitope is contained in the top K predictions for an individual TCR.

##### Average rank

In the same setting as for R@K, the set of epitopes is ordered by prediction value. The Average Rank corresponds to the mean position of the cognate epitope within this order over all TCRs.

##### Pearson and Spearman correlation

While the other metrics evaluate the predictors’ classification capability, correlation-based metrics indicate whether the prediction score is also quantitatively associated with TCR binding or activation. While the Pearson coefficient measures the linear relationship between prediction and the true score, the Spearman rank coefficient describes their potentially non-linear monotonic relationship, i.e., comparing the orders within both scores.

Metrics computed over the full dataset cannot be competitively compared between general and categorical models as they depend on all epitopes. Further, no categorical models exist for the epitopes contained in the mutational dataset. We used a metric score resembling random prediction in the case of epitopes for which categorical methods did not provide a model. For classification metrics, the AUC was assigned a value of 0.5, and APS and F1-Score the frequency of the epitope in the dataset. As unavailable classes can never be detected in repertoire annotation, we assumed an R@K value of 0.0 and an average rank of equal to the mean of the maximum rank and the lowest unassigned rank (i.e., the first rank beyond the available models). This negative effect for unobserved classes was balanced by the reduced number of candidates for available epitopes.

### Quantification and statistical analysis

All statistical analyses were performed in Python (version 3.8.20) using the libraries SciPy (version 1.10.1), Numpy (version 1.24.3), and Pandas (version 2.0.3). The sample size n and its description can be found in the corresponding figure legends. All boxplots indicate the data quartiles while the whiskers extend to the extreme values, excluding outliers outside the 1.5 interquartile range. The median is indicated as a horizontal line. Regression plots show the linear regression fit indicating the 95% confidence interval as an error band. Bar plots represent the data mean and their error bars the 95% confidence interval. Significance was defined by a *p*-value less than 0.05. For Spearman and Pearson correlations, a t-test against the null hypothesis that the data is uncorrelated was performed. The present study utilized all samples, excluding data points described in the Method section.

## Supplemental information

Document S1. Figures S1–S12 and Tables S1–S6

Data S1. Benchmarking results on the viral dataset, related to Figure 2 All metric scores (AUC, APS, F1-Score, average Rank, R@1, and R@3) on the full data, macro-averaged, averaged, and per epitope

Data S2. Predictions of the viral dataset, related to Figure 2 Prediction score for each method between all combinations of epitopes and TCRs of the viral dataset

Data S3. Benchmarking results on the mutation dataset, related to Figure 3 All metric scores (AUC, APS, F1-Score, Pearson correlation, and Spearman coefficient) on the full data, macro-averaged, averaged, and per TCR

Data S4. Predictions of the mutation dataset, related to Figure 3 Prediction score for each method between all combinations of epitopes and TCRs of the mutation dataset

Data S5. Benchmarking results on the murine mutation dataset, related to Figure 3 All metric scores (AUC, APS, F1-Score, Pearson correlation, and Spearman coefficient) on the full data, macro-averaged, averaged, and per TCR for applicable methods

Data S6. Predictions of the murine mutation dataset, related to Figure 3 Prediction score for applicable methods between all combinations of epitopes and TCRs of the mutation dataset

Document S2. Article plus supplemental information

## References

[1] Springer I., Tickotsky N., Louzoun Y. (2021). Contribution of t cell receptor alpha and beta cdr3, mhc typing, v and j genes to peptide binding prediction. Front. Immunol..

[2] Davis M.M. (2020). T cell analysis in vaccination. Curr. Opin. Immunol..

[3] D’Ippolito E., Wagner K.I., Busch D.H. (2020). Needle in a haystack: the naïve repertoire as a source of t cell receptors for adoptive therapy with engineered t cells. Int. J. Mol. Sci..

[4] Serr I., Drost F., Schubert B., Daniel C. (2021). Antigen-specific treg therapy in type 1 diabetes- challenges and opportunities. Front. Immunol..

[5] Scott D., Singer D.S. (2023). Harnessing the power of discovery. Cancer Discov..

[6] 10x Genomics (2019). A new way of exploring immunity-linking highly multiplexed antigen recognition to immune repertoire and phenotype. Tech. Rep..

[7] Dash P., Fiore-Gartland A.J., Hertz T., Wang G.C., Sharma S., Souquette A., Crawford J.C., Clemens E.B., Nguyen T.H.O., Kedzierska K. (2017). Quantifiable predictive features define epitope-specific t cell receptor repertoires. Nature.

[8] Glanville J., Huang H., Nau A., Hatton O., Wagar L.E., Rubelt F., Ji X., Han A., Krams S.M., Pettus C. (2017). Identifying specificity groups in the t cell receptor repertoire. Nature.

[9] Chronister W.D., Crinklaw A., Mahajan S., Vita R., Koşaloğlu-Yalçın Z., Yan Z., Greenbaum J.A., Jessen L.E., Nielsen M., Christley S. (2021). Tcrmatch: predicting t-cell receptor specificity based on sequence similarity to previously characterized receptors. Front. Immunol..

[10] Sidhom J.-W., Larman H.B., Pardoll D.M., Baras A.S. (2021). Deeptcr is a deep learning framework for revealing sequence concepts within t-cell repertoires. Nat. Commun..

[11] Wu K., Yost K.E., Daniel B., Belk J.A., Xia Y., Egawa T., Satpathy A., Chang H.Y., Zou J. (2021). Tcr-bert: learning the grammar of t-cell receptors for flexible antigen-xbinding analyses. bioRxiv.

[12] Drost F., Schiefelbein L., Schubert B. (2022). metcrs-learning a metric for t-cell receptors. bioRxiv.

[13] Vita R., Mahajan S., Overton J.A., Dhanda S.K., Martini S., Cantrell J.R., Wheeler D.K., Sette A., Peters B. (2019). The immune epitope database (iedb): 2018 update. Nucleic Acids Res..

[14] Bagaev D.V., Vroomans R.M.A., Samir J., Stervbo U., Rius C., Dolton G., Greenshields-Watson A., Attaf M., Egorov E.S., Zvyagin I.V. (2020). Vdjdb in 2019: database extension, new analysis infrastructure and a t-cell receptor motif compendium. Nucleic Acids Res..

[15] Tickotsky N., Sagiv T., Prilusky J., Shifrut E., Friedman N. (2017). Mcpas-tcr: a manually curated catalogue of pathology-associated t cell receptor sequences. Bioinformatics.

[16] Jokinen E., Huuhtanen J., Mustjoki S., Heinonen M., Lähdesmäki H. (2021). Predicting recognition between t cell receptors and epitopes with tcrgp. PLoS Comput. Biol..

[17] Jurtz V.I., Jessen L.E., Bentzen A.K., Jespersen M.C., Mahajan S., Vita R., Jensen K.K., Marcatili P., Hadrup S.R., Peters B., Nielsen M. (2021). Nettcr: sequence-based prediction of tcr binding to peptide-mhc complexes using convolutional neural networks. bioRxiv.

[18] Croce G., Bobisse S., Moreno D.L., Schmidt J., Guillame P., Harari A., Gfeller D. (2024). Deep learning predictions of tcr-epitope interactions reveal epitope-specific chains in dual alpha t cells. Nat. Commun..

[19] Springer I., Besser H., Tickotsky-Moskovitz N., Dvorkin S., Louzoun Y. (2020). Prediction of specific tcr-peptide binding from large dictionaries of tcr-peptide pairs. Front. Immunol..

[20] Moris P., De Pauw J., Postovskaya A., Gielis S., De Neuter N., Bittremieux W., Ogunjimi B., Laukens K., Meysman P. (2021). Current challenges for unseen-epitope tcr interaction prediction and a new perspective derived from image classification. Brief. Bioinform..

[21] Fischer D.S., Wu Y., Schubert B., Theis F.J. (2020). Predicting antigen specificity of single t cells based on tcr cdr 3 regions. Mol. Syst. Biol..

[22] Deng L., Ly C., Abdollahi S., Zhao Y., Prinz I., Bonn S. (2023). Performance comparison of tcr-pmhc prediction tools reveals a strong data dependency. Front. Immunol..

[23] Dens C., Laukens K., Bittremieux W., Meysman P. (2023). The pitfalls of negative data bias for the t-cell epitope specificity challenge. bioRxiv.

[24] Meysman P., Barton J., Bravi B., Cohen-Lavi L., Karnaukhov V., Lilleskov E., Montemurro A., Nielsen M., Mora T., Pereira P. (2023). Benchmarking solutions to the t-cell receptor epitope prediction problem: Immrep22 workshop report. ImmunoInformatics.

[25] Barton, J. (2023). Immrep23: Tcr specificity prediction challenge.

[26] Nielsen M., Eugster A., Jensen M.F., Goel M., Tiffeau-Mayer A., Pelissier A., Valkiers S., Martínez M.R., Meynard-Piganeeau B., Greiff V. (2024). Lessons learned from the immrep23 tcr-epitope prediction challenge. ImmunoInformatics.

[27] Schubert B., Walzer M., Brachvogel H.P., Szolek A., Mohr C., Kohlbacher O. (2016). Fred 2: an immunoinformatics framework for python. Bioinformatics.

[28] Kocher K., Drost F., Tesfaye A.M., Moosmann C., Schuelein C., Grotz M., D'Ippolito E., Graw F., Spriewald B., Busch D.H. (2024). Quality of vaccination-induced t cell responses is conveyed by polyclonality and high, but not maximum, antigen receptor avidity. bioRxiv.

[29] Habern, O. (2022). Introducing beam (barcode enabled antigen mapping): Benefits of rapid, antigen-specific b-and t-cell discovery. 10x Genomics.

[30] Drost F., Dorigatti E., Straub A., Hilgendorf P., Wagner K.I., Heyer K., López Montes M., Bischl B., Busch D.H., Schober K., Schubert B. (2024). Predicting t cell receptor functionality against mutant epitopes. Cell Genom..

[31] Vander Heiden J.A., Marquez S., Marthandan N., Bukhari S.A.C., Busse C.E., Corrie B., Hershberg U., Kleinstein S.H., Matsen Iv F.A., Ralph D.K. (2018). Airr community standardized representations for annotated immune repertoires. Front. Immunol..

[32] Sturm G., Szabo T., Fotakis G., Haider M., Rieder D., Trajanoski Z., Finotello F. (2020). Scirpy: a scanpy extension for analyzing single-cell t-cell receptor-sequencing data. Bioinformatics.

[33] Cai M., Bang S., Zhang P., Lee H. (2022). Atm-tcr: Tcr-epitope binding affinity prediction using a multi-head self-attention model. Front. Immunol..

[34] Xu Y., Qian X., Tong Y., Li F., Wang K., Zhang X., Liu T., Wang J. (2022). Attntap: A dual-input framework incorporating the attention mechanism for accurately predicting tcr-peptide binding. Front. Genet..

[35] Myronov A., Mazzocco G., Krol P., Plewczynski D. (2023). Bertrand-peptide: Tcr binding prediction using bidirectional encoder representations from transformers augmented with random tcr pairing. bioRxiv.

[36] Xu Z., Luo M., Lin W., Xue G., Wang P., Jin X., Xu C., Zhou W., Cai Y., Yang W. (2021). Dlptcr: an ensemble deep learning framework for predicting immunogenic peptide recognized by t cell receptor. Brief. Bioinform..

[37] Pham M.-D.N., Nguyen T.N., Tran L.S., Nguyen Q.T.B., Nguyen T.P.H., Pham T.M.Q., Nguyen H.N., Giang H., Phan M.D., Nguyen V. (2023). epitcr: a highly sensitive predictor for tcr-peptide binding. Bioinformatics.

[38] Zhang Y., Jian X., Xu L., Zhao J., Lu M., Lin Y., Xie L. (2023). itcep: a deep learning framework for identification of t cell epitopes by harnessing fusion features. Front. Genet..

[39] Jensen M.F., Nielsen M. (2023). Nettcr 2.2-improved tcr specificity predictions by combining pan-and peptide-specific training strategies, loss-scaling and integration of sequence similarity. bioRxiv.

[40] Gao Y., Gao Y., Fan Y., Zhu C., Wei Z., Zhou C., Chuai G., Chen Q., Zhang H., Liu Q. (2023). Pan-peptide meta learning for t-cell receptor-antigen binding recognition. Nat. Mach. Intell..

[41] Lu T., Zhang Z., Zhu J., Wang Y., Jiang P., Xiao X., Bernatchez C., Heymach J.V., Gibbons D.L., Wang J. (2021). Deep learning-based prediction of the t cell receptor-antigen binding specificity. Nat. Mach. Intell..

[42] Kwee B.P.Y., Messemaker M., Marcus E., Oliveira G., Scheper W., Wu C.J., Teuwen J., Schumacher T.N. (2023). Stapler: Efficient learning of tcr-peptide specificity prediction from full-length tcr-peptide data. bioRxiv.

[43] Peng X., Lei Y., Feng P., Jia L., Ma J., Zhao D., Zeng J. (2023). Characterizing the interaction conformation between t-cell receptors and epitopes with deep learning. Nat. Mach. Intell..

[44] Jiang Y., Huo M., Cheng Li S. (2023). Teinet: a deep learning framework for prediction of tcr- epitope binding specificity. Brief. Bioinform..

[45] Weber A., Born J., Rodriguez Martínez M. (2021). Titan: T-cell receptor specificity prediction with bimodal attention networks. Bioinformatics.

[46] Meynard-Piganeau B., Feinauer C., Weigt M., Walczak A.M., Mora T. (2023). Tulip-a transformer based unsupervised language model for interacting peptides and t-cell receptors that generalizes to unseen epitopes. bioRxiv.

[47] Weininger D., Weininger A., Weininger J.L. (1989). Smiles. 2. algorithm for generation of unique smiles notation. J. Chem. Inf. Comput. Sci..

[48] Zhang W., Wang L., Liu K., Wei X., Yang K., Du W., Wang S., Guo N., Ma C., Luo L. (2020). Pird: pan immune repertoire database. Bioinformatics.

[49] Francis J.M., Leistritz-Edwards D., Dunn A., Tarr C., Lehman J., Dempsey C., Hamel A., Rayon V., Liu G., Wang Y. (2022). Allelic variation in class i hla determines cd8+ t cell repertoire shape and cross-reactive memory responses to sars-cov-2. Sci. Immunol..

[50] Jensen M.F., Nielsen M. (2024). Enhancing tcr specificity predictions by combined pan-and peptide-specific training, loss-scaling, and sequence similarity integration. eLife.

[51] Grazioli F., Mösch A., Machart P., Li K., Alqassem I., O'Donnell T.J., Min M.R. (2022). On tcr binding predictors failing to generalize to unseen peptides. Front. Immunol..

[52] Heather J.M., Spindler M.J., Alonso M.H., Shui Y.I., Millar D.G., Johnson D.S., Cobbold M., Hata A.N. (2022). Stitchr: stitching coding tcr nucleotide sequences from v/j/cdr3 information. Nucleic Acids Res..

[53] Dunbar J., Deane C.M. (2016). Anarci: antigen receptor numbering and receptor classification. Bioinformatics.

[54] Kocher K., Moosmann C., Drost F., Schülein C., Irrgang P., Steininger P., Zhong J., Träger J., Spriewald B., Bock C., Busch D.H. (2024). Adaptive immune responses are larger and functionally preserved in a hypervaccinated individual. Lancet Infect. Dis..

[55] Straub A., Grassmann S., Jarosch S., Richter L., Hilgendorf P., Hammel M., Wagner K.I., Buchholz V.R., Schober K., Busch D.H. (2023). Recruitment of epitope-specific t cell clones with a low-affinity threshold supports efficacy against mutational escape upon re-infection. Immunity.

[56] Oh Song H., Xiang Y., Jegelka S., Savarese S. (2016). Proceedings of the IEEE conference on computer vision and pattern recognition.

