# Supplementary material for: Benchmarking of T cell receptor-epitope predictors with ePytope-TCR
Source: Cell Genom. 2025 Jul 7;5(8):100946. doi: 10.1016/j.xgen.2025.100946 (PMC12366652; doi:10.1016/j.xgen.2025.100946)
Supplement: Document S1. Figures S1–S12 and Tables S1–S6 [file mmc1.pdf]

Cell Genomics, Volume *5*

Supplemental information

Benchmarking of T cell receptor-epitope predictors with ePytope-TCR

Felix Drost, Anna Chernysheva, Mahmoud Albahah, Katharina Kocher, Kilian Schober, and Benjamin Schubert

Supplementary Figures

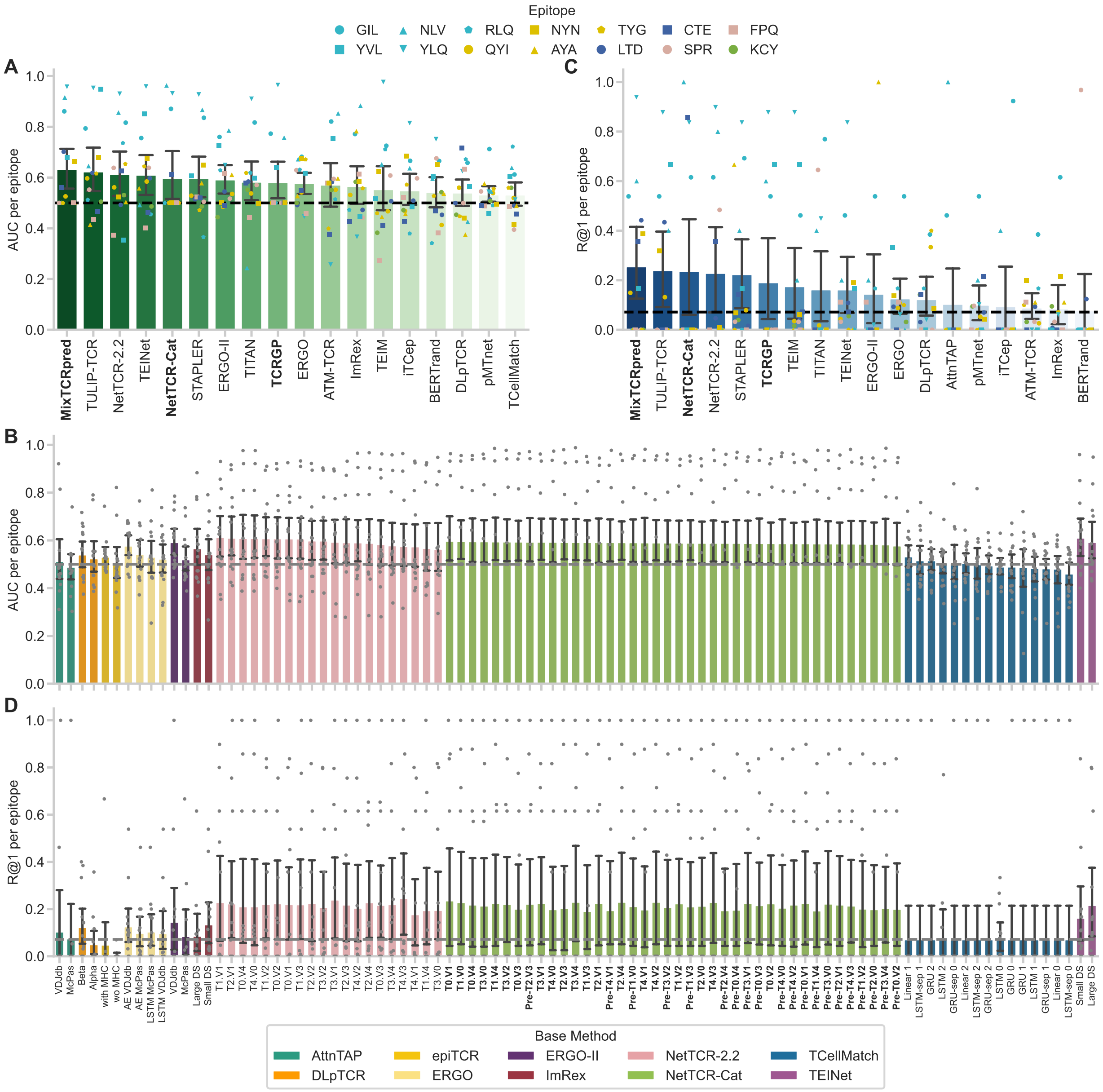

Figure S1 | Performance on the viral dataset, related to Figure 2. Performance scores of all models on the viral benchmark for AUC (**A**) and R@1 (**B**). The models are ordered by their performance on the respective metric. Performance scores of methods with multiple model versions on the viral benchmark for AUC (**C**) and R@1 (**D**). The models are ordered by their performance on the respective metric within their method. Categorical predictors are marked with boldface names. The mean indicates the average metric score measured for each epitope individually, while the error bars indicate the 95% confidence interval over the scores ($n = 14$ epitopes). The dashed black line marks random predictions.

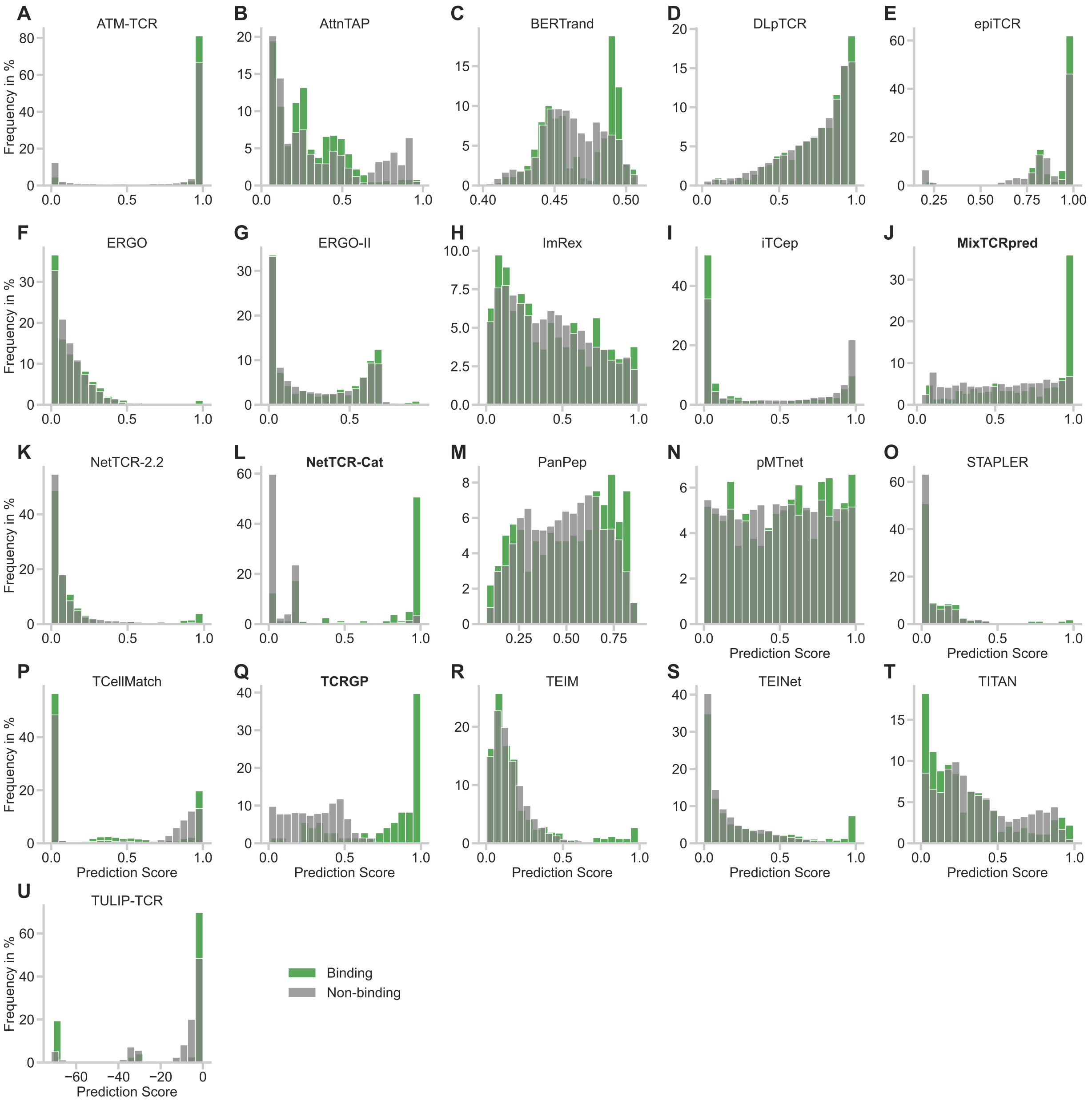

Figure S2 | Distribution of prediction scores on the viral dataset, related to Figure 2. Binding score frequency for $n = 20$ bins between positive and negative pairs for all models (**A-U**). Categorical models are marked with boldface names.

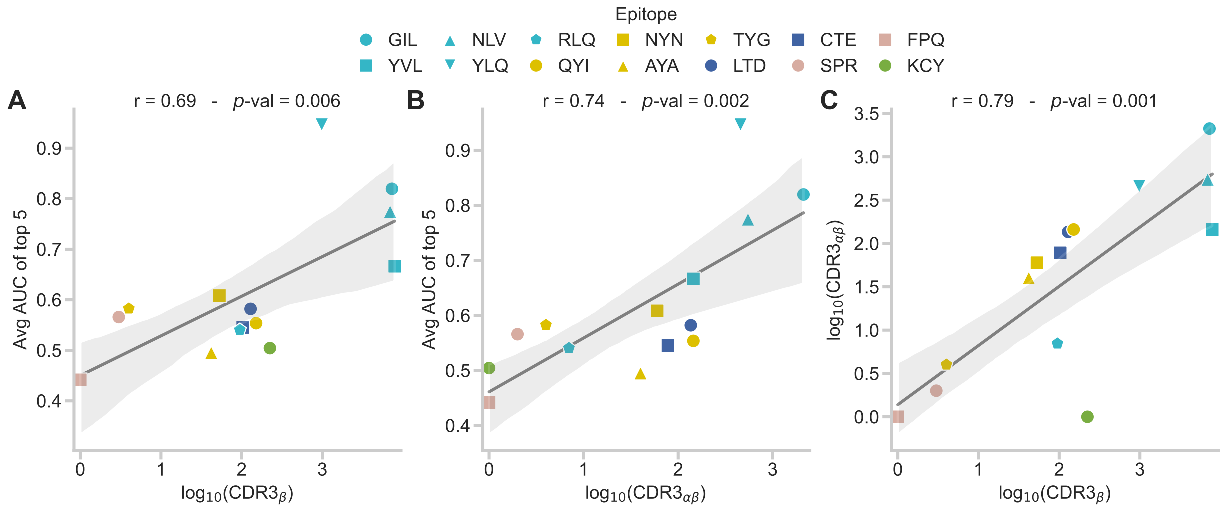

Figure S3 | Dependency of model performance on data availability, related to Figure 2. Pearson correlation between the average AUC score of the best five predictors and the amount of unique pairs between epitopes and the CDR3β (**A**) or $\alpha\beta$-paired CDR3 sequences (**B**) available in public databases for all epitopes ($n = 14$). **C**, Correlation between the amount of publicly available CDR3β and $\alpha\beta$-paired CDR3 sequences across all epitopes ($n = 14$).

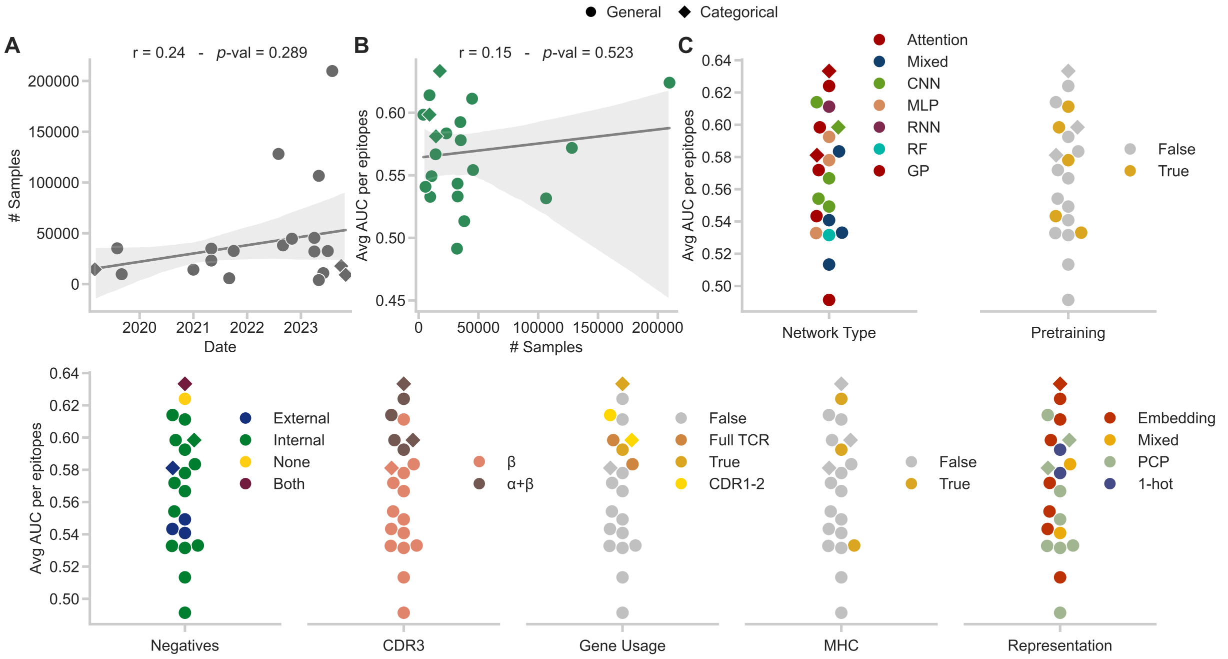

Figure S4 | Impact of model properties on the performance on the viral dataset, related to Figure 2. A, Pearson correlation between the number of positive training samples and the initial publication date of the methods ($n = 21$). **B,** Pearson correlation between the average AUC score of the predictors and the number of positive training samples ($n = 21$). **C,** Average AUC indicated for different method design choices.

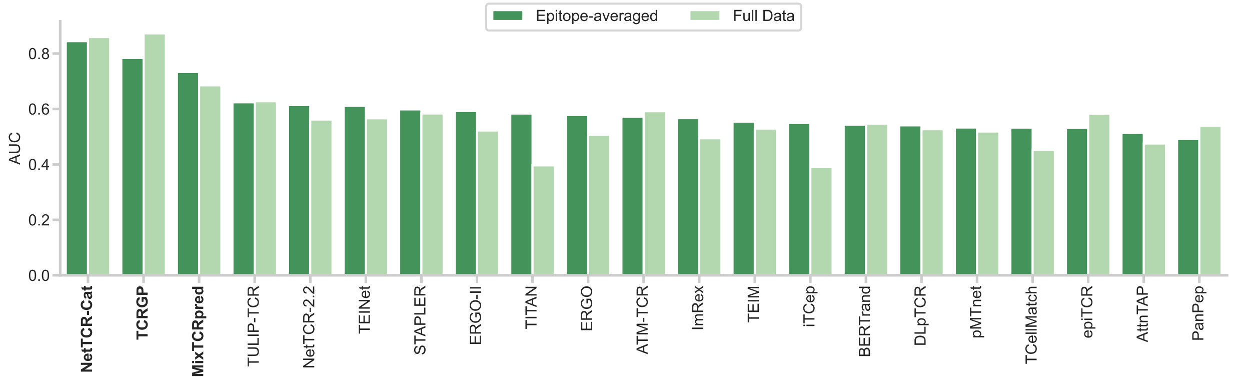

Figure S5 | Differences in AUC calculation on the viral dataset, related to Figure 2. Comparison between the AUC averaged per epitope ($n = 14$) and the AUC calculated on the full dataset. The models are ordered by best averaged AUC. Categorical predictors are marked with boldface names. Note, that for categorical methods the AUC was calculated only on epitopes with available models and, therefore, is not comparable to other predictors.

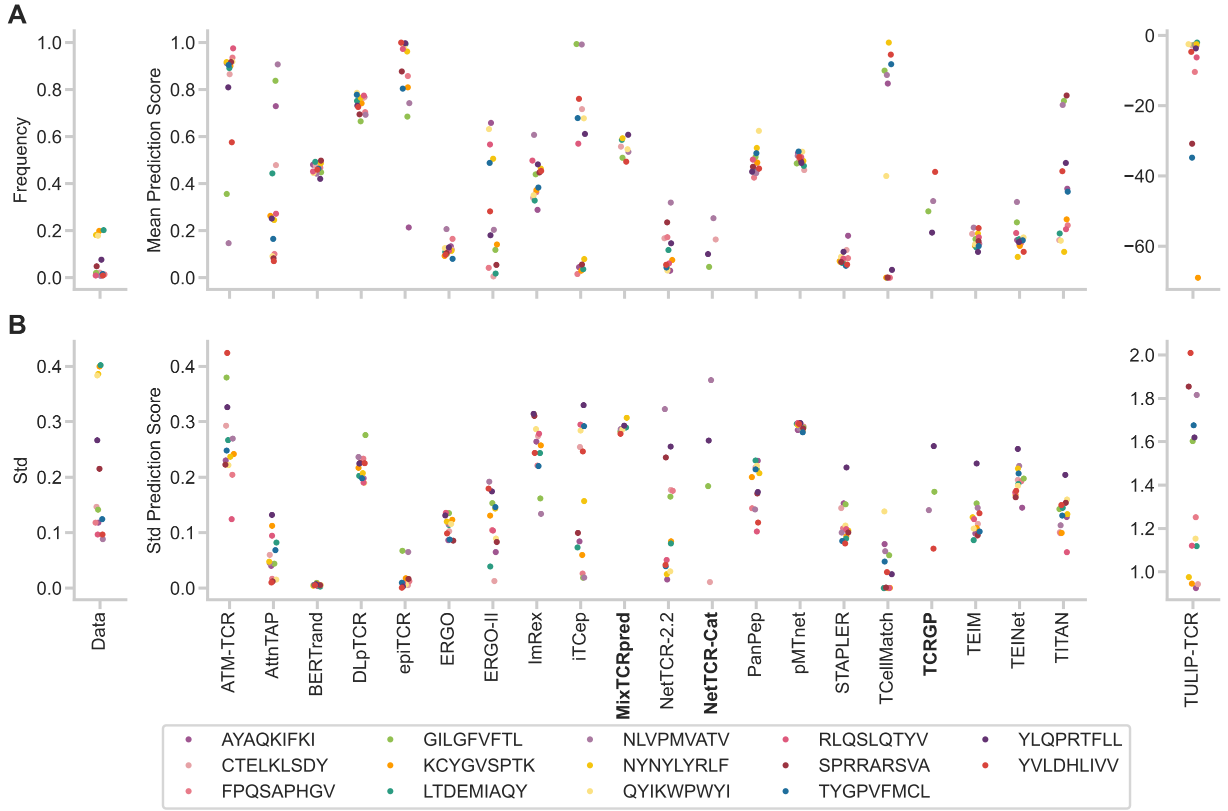

Figure S6 | Prediction scores per epitope on the viral dataset, related to Figure 2. True data distribution and prediction scores statistics over all TCRs ($n = 638$) for each epitope indicating the mean (**A**) and standard deviation (**B**). Categorical predictors are marked with boldface names.

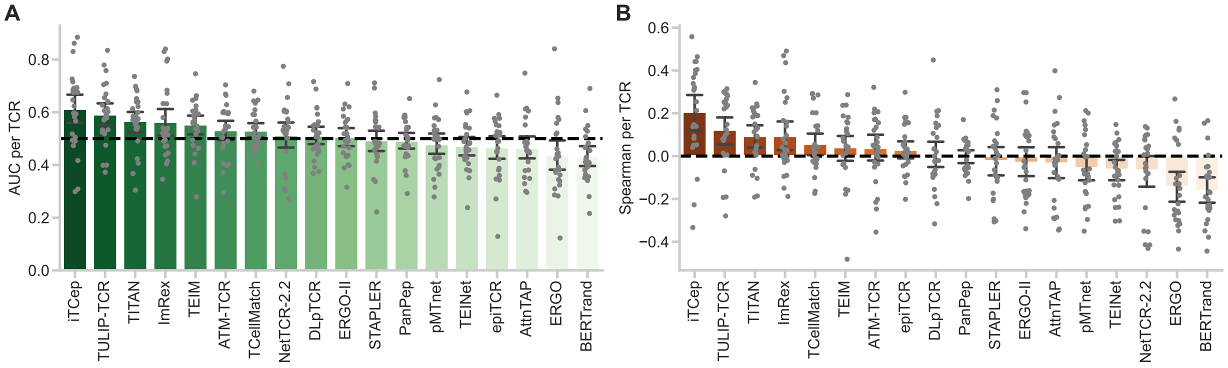

Figure S7 | Performance of all models on the mutation dataset, related to Figure 3. Performance scores of all models on the mutation benchmark for AUC (**A**) and Spearman coefficient (**B**). The models are ordered by their performance on the respective metric. The mean indicates the average metric score measured for each epitope individually, while the error bars indicate the 95% confidence interval over the scores ($n = 26$ TCRs). The dashed black line marks random predictions.

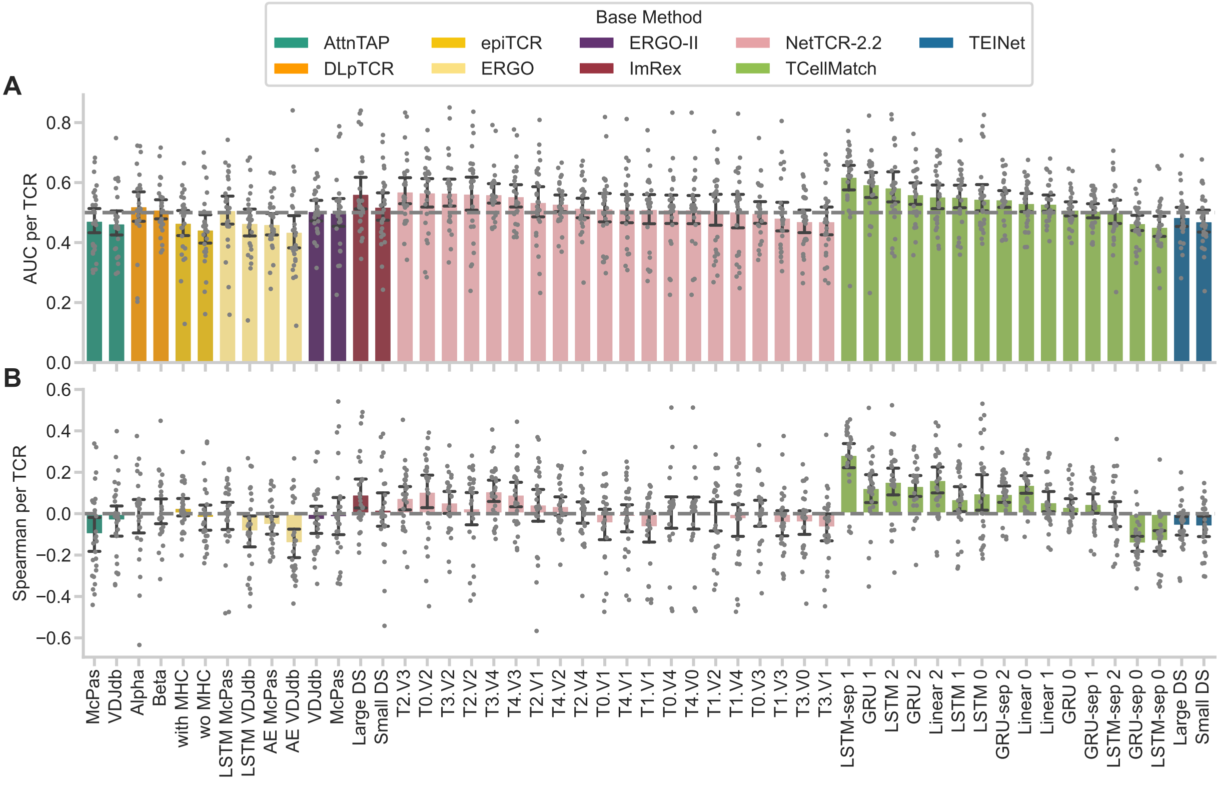

Figure S8 | Performance of model alternatives on the mutation dataset, related to Figure 3. Performance scores of methods with multiple model versions on the mutation benchmark for AUC (**A**) and R@1 (**B**). The models are ordered by their performance on the respective metric within their method. The mean indicates the average metric score measured for each epitope individually, while the error bars indicate the 95% confidence interval over the scores ($n = 26$ TCRs). The dashed black line marks random predictions.

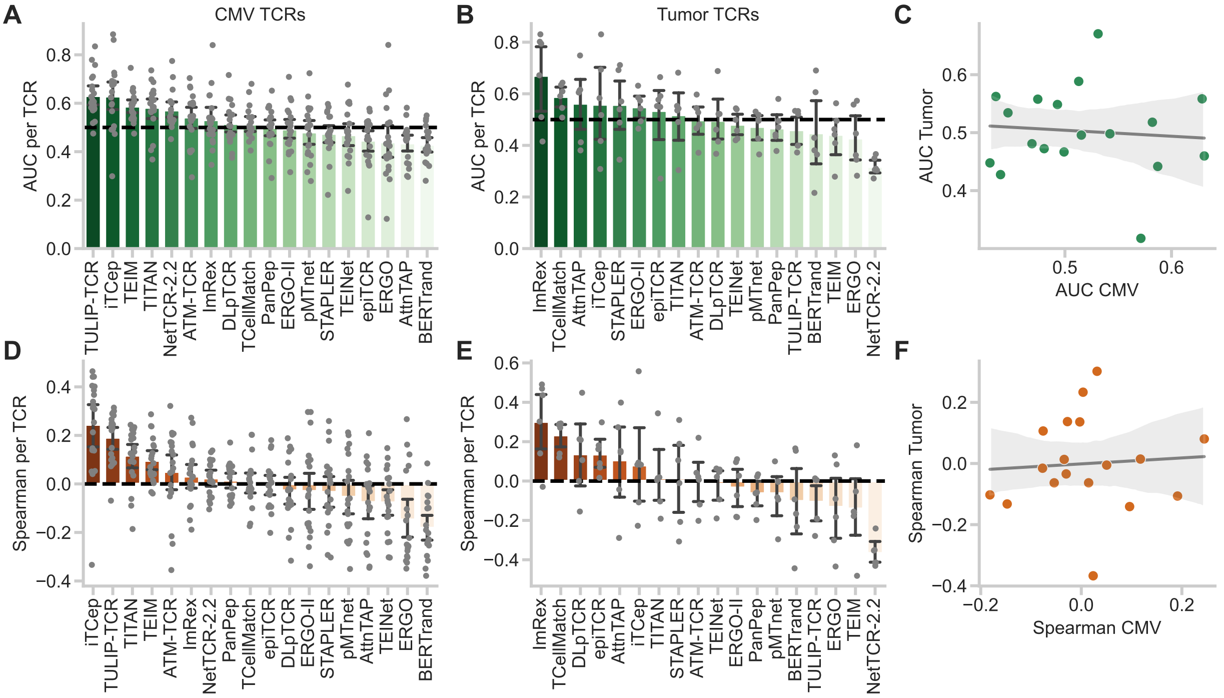

Figure S9 | Separate performance on the two subsets of the mutation dataset, related to Figure 3. Performance scores of all methods on the CMV (left column) and Tumor TCRs (middle column) of the mutation benchmark for AUC (**A**, **B**) and Spearman correlation (**D**, **E**) calculated per TCR. The models are ordered by their performance on the respective metric. The mean indicates the average metric score measured for each epitope individually, while the error bars indicate the 95% confidence interval over the scores (n = 26 TCRs). The dashed black line marks random predictions. Correlation of all methods (n = 18) between the performance on the CMV and tumor TCRs for AUC (**C**) and Spearman correlation (**F**).

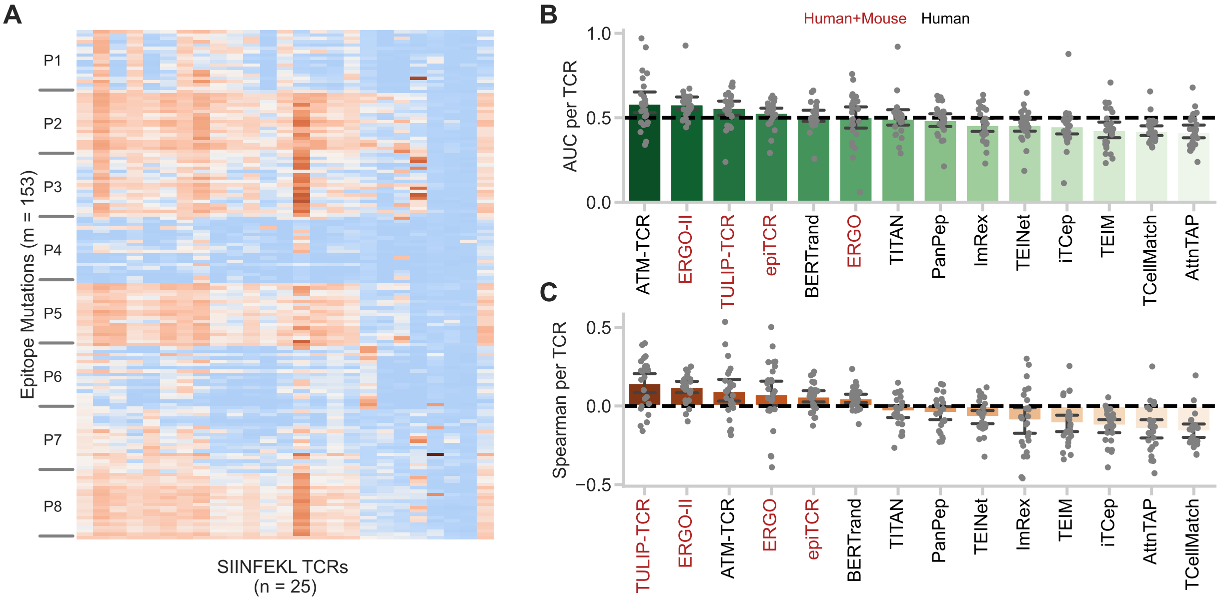

Figure S10 | Benchmark on TCRs reactivity against mutations of a murine epitope, related to Figure 3. **A**, The dataset consists of a deep mutational scan of TCRs reactive to mutations of the murine model epitope SIINFEKL. Prediction performance for this dataset is shown for all predictors, measured by the Area Under the Curve (AUC, **B**) and Spearman correlation coefficient (Spearman, **C**) calculated per TCR. The mean indicates the average metric score measured for each TCR individually, while the error bars indicate the 95% confidence interval over the scores (n = 25 TCRs). Models trained on murine and human TCRs are highlighted in red.

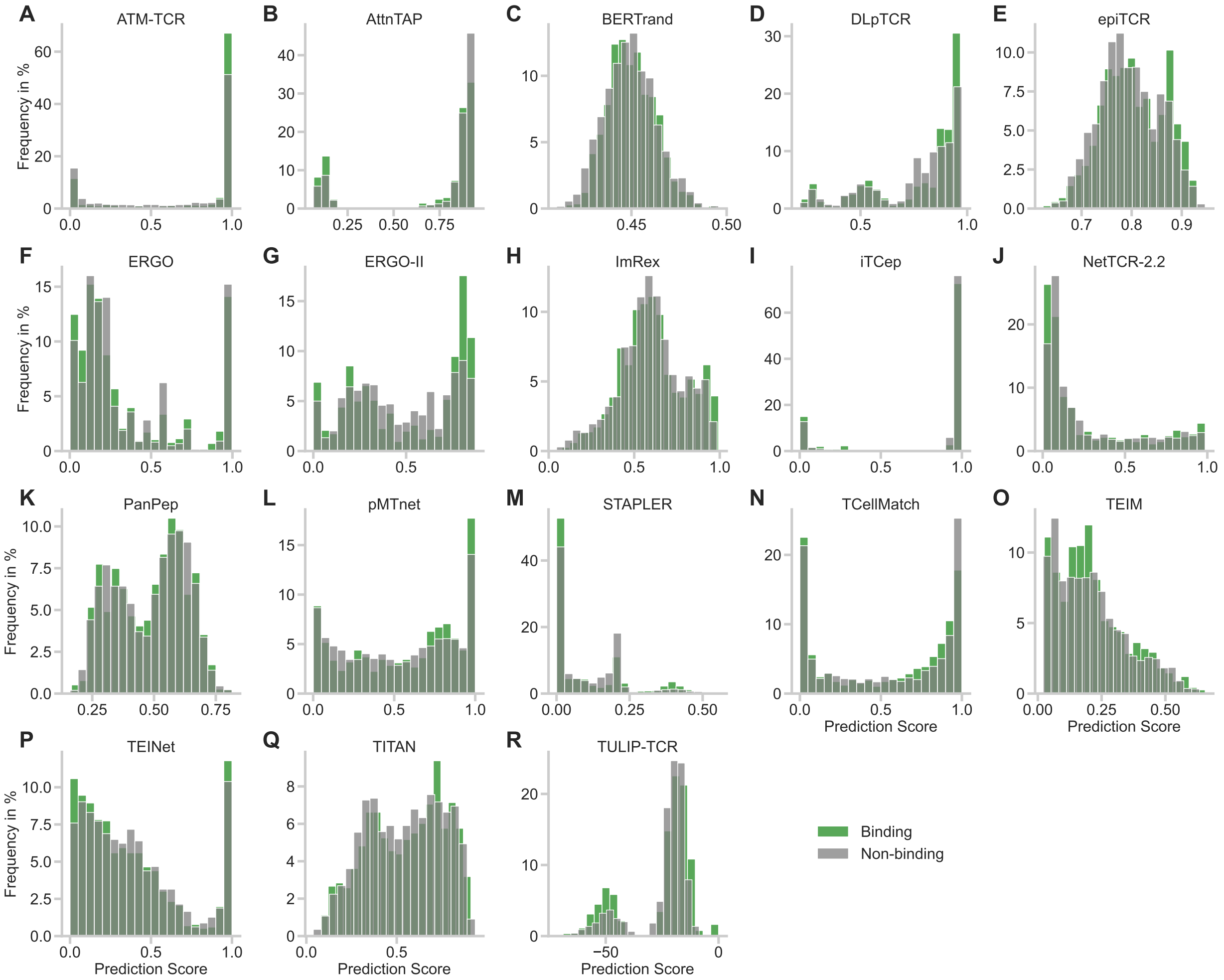

Figure S11 | Distribution of prediction scores on the mutation dataset, related to Figure 3. Binding score frequency for $n = 20$ bins between positive and negative pairs for all models (**A-R**).

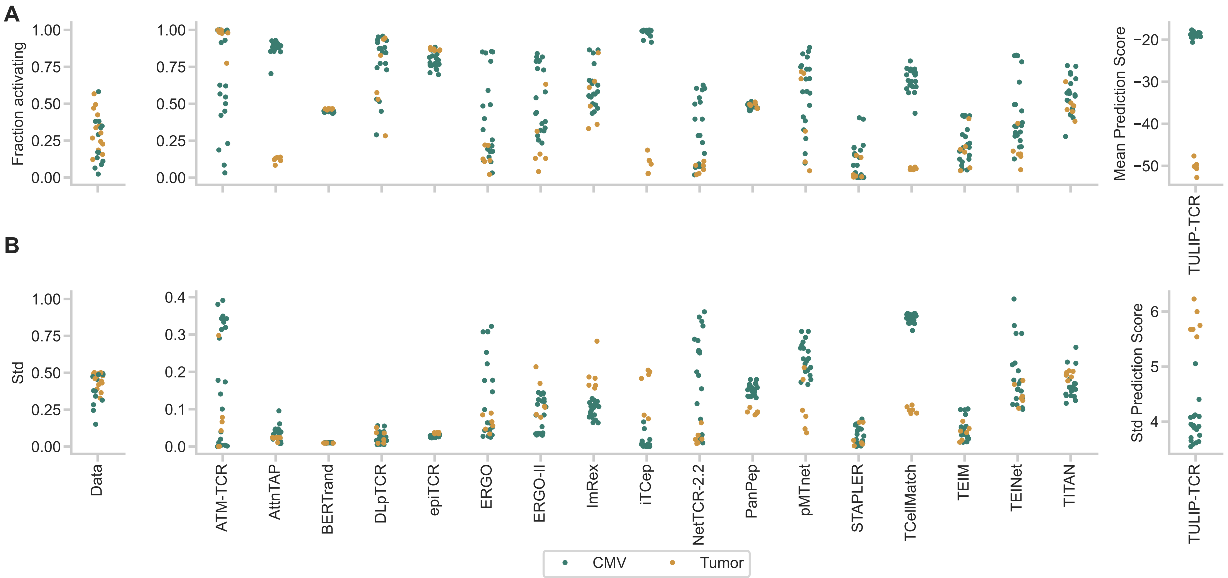

Figure S12 | Prediction scores per TCR on the mutation dataset, related to Figure 3. True data distribution and prediction scores statistics over all epitopes ($n = 134$ for tumor TCRs, $n = 172$ for CMV TCRs) for each TCR indicating the mean (**A**) and standard deviation (**B**).

Supplementary Tables

Name	Authors	Date	License
ATM-TCR	Cai et al.	07/2022	Attribution 4.0 International
AttnTAP	Xu et al.	08/2022	Custom, non-commercial
BERTrand	Myronov et al.	06/2023	MIT
DLpTCR	Xu et al.	08/2021	Custom, non-commercial
epiTCR	Pham et al.	04/2023	MIT
ERGO	Springer et al.	07/2019	MIT
ERGO-II	Springer et al.	04/2021	MIT
ImRex	Moris et al.	12/2020	Custom, non-commercial
iTCep	Zhang et al.	05/2023	GNU
MixTCRpred	Croce et al.	09/2023	Custom, non-commercial
NetTCR-2.2	Jensen et al.	10/2023	Custom, non-commercial
NetTCR-Cat	Jensen et al.	10/2023	Custom, non-commercial
PanPep	Gao et al.	03/2023	GNU
pMTnet	Lu et al.	09/2021	GNU
STAPLER	Kwee et al.	04/2023	Apache License
TCellMatch	Fischer et al.	08/2019	BSD 3-Clause
TCRGP	Jokinen et al.	02/2019	MIT
TEIM	Peng et al.	03/2023	MIT
TEINet	Jiang et al.	10/2022	GNU
TITAN	Weber et al.	04/2021	Custom
TULIP-TCR	Meynard-Piganeau et al.	07/2023	GNU

Table S1 | Additional predictor information, related to Table 1. For each supported tool the name, the code source, first author, publication date, and license of the model is provided. The code repository of each predictor is linked via the name. The first occurrence in a journal, conference, or preprint is considered as the publication date.

	Full Data						Per Epitope					
	AUC	APS	F1-Score	Rank	R@1	R@3	AUC	APS	F1-Score	Rank	R@1	R@3
ATM-TCR	**0.59**	0.09	0.16	**6.28**	0.08	0.28	0.57±0.16	0.13±0.13	0.15±0.14	6.86±2.01	0.09±0.11	0.26±0.18
AttnTAP	0.48	0.07	0.14	7.80	0.02	0.04	0.51±0.16	0.12±0.16	0.10±0.13	7.29±4.20	0.10±0.29	0.21±0.43
BERTrand	0.55	0.09	**0.21**	6.92	0.07	**0.43**	0.54±0.12	0.12±0.13	0.06±0.13	7.35±4.05	0.08±0.26	0.21±0.42
DLpTCR	0.53	0.08	0.14	**6.20**	0.13	0.30	0.54±0.11	0.08±0.08	0.13±0.13	6.85±2.40	0.12±0.15	0.25±0.21
epiTCR	0.58	0.08	0.17	6.46	0.01	0.18	0.53±0.07	0.07±0.08	0.08±0.13	7.46±4.13	0.05±0.18	0.20±0.35
ERGO	0.51	0.09	0.13	7.45	0.09	0.25	0.58±0.09	0.13±0.14	0.13±0.12	6.36±1.71	0.13±0.15	0.32±0.15
ERGO-II	0.52	0.09	0.14	7.35	0.03	0.28	0.59±0.11	0.13±0.12	0.13±0.14	6.78±4.00	0.15±0.29	0.30±0.36
ImRex	0.49	0.08	0.13	7.68	0.09	0.24	0.57±0.15	0.12±0.12	0.12±0.13	7.12±2.09	0.09±0.16	0.28±0.26
iTCep	0.39	0.05	0.13	8.93	0.02	0.10	0.55±0.12	0.11±0.14	0.12±0.13	7.23±3.93	0.09±0.26	0.25±0.37
**MixTCRpred**	0.69*	0.40*	0.40*	3.18*	0.41*	0.61*	**0.63±0.17**	**0.25±0.28**	**0.23±0.26**	6.51±4.54	**0.26±0.30**	**0.40±0.39**
NetTCR-2.2	0.56	0.11	0.15	7.02	0.13	0.23	**0.61±0.17**	0.17±0.22	0.14±0.13	**5.97±3.05**	0.23±0.33	0.34±0.38
**NetTCR-Cat**	0.86*	0.35*	0.44*	1.35*	0.81*	0.95*	0.60±0.18	0.18±0.24	**0.16±0.21**	7.17±3.83	**0.24±0.40**	0.27±0.44
PanPep	0.54	0.09	0.16	6.59	0.13	**0.32**	0.49±0.10	0.09±0.12	0.09±0.12	7.60±2.60	0.07±0.19	0.20±0.27
pMTnet	0.52	0.08	0.14	7.27	0.08	0.24	0.53±0.06	0.10±0.10	0.12±0.12	6.93±1.06	0.10±0.14	0.28±0.12
STAPLER	**0.58**	**0.15**	0.16	6.42	0.13	0.31	0.60±0.17	0.19±0.25	0.15±0.14	**5.90±2.19**	0.22±0.30	0.37±0.32
TCellMatch	0.45	0.08	0.18	8.11	**0.18**	0.21	0.53±0.09	0.08±0.08	0.02±0.08	7.50±4.18	0.07±0.27	0.21±0.43
**TCRGP**	0.87*	0.68*	0.74*	1.38*	0.78*	0.97*	0.58±0.15	0.18±0.23	**0.20±0.26**	7.22±3.75	0.19±0.32	0.28±0.46
TEIM	0.53	0.14	0.15	7.03	0.12	0.26	0.55±0.18	0.17±0.24	0.12±0.22	6.25±3.00	0.18±0.28	**0.38±0.36**
TEINet	0.57	**0.19**	**0.19**	6.81	**0.16**	0.32	0.61±0.16	**0.20±0.24**	0.16±0.22	**6.04±2.46**	0.16±0.23	0.36±0.24
TITAN	0.40	0.07	0.13	8.88	0.09	0.16	0.58±0.15	0.15±0.17	0.12±0.13	6.71±3.78	0.16±0.28	0.29±0.44
TULIP-TCR	**0.63**	**0.25**	**0.26**	**6.00**	**0.26**	**0.44**	**0.62±0.17**	**0.22±0.26**	0.15±0.24	6.42±4.57	**0.24±0.30**	**0.38±0.38**

Table S2 | Performance on the viral dataset, related to Figure 2. The reported values correspond to the performance evaluated on either the full dataset or per epitope for classification (AUC: Area under the receiver operating characteristic curve, APS: average precision score, F1-Score) or ranking (Rank: Average rank of the correct epitope, R@K: Recall at k). Categorical models are marked with boldface method names. The mean performance and standard deviation are indicated when the performance was evaluated per epitope ($n = 14$ epitopes). The three best scores for each metric are highlighted in boldface. Metric scores marked with an asterisk are not comparable between models as they could not be calculated across the full dataset and are, therefore, excluded from the highlighted best models.

	High					Medium								Low			
	YVL	GIL	NLV	YLQ	Avg	KCY	QYI	LTD	CTE	RLQ	NYN	AYA	Avg	TYG	SPR	FPQ	Avg
ATM-TCR	**0.75**	0.82	0.85	0.26	0.67	0.55	0.55	0.58	0.37	0.51	0.58	0.60	0.54	0.40	0.59	0.58	0.52
AttnTAP	0.39	0.81	0.31	0.92	0.61	0.42	0.46	0.49	0.49	0.46	0.46	0.44	0.46	0.56	0.48	0.49	0.51
BERTrand	0.60	0.67	0.60	0.75	0.65	0.54	0.41	0.45	0.47	0.34	0.65	0.54	0.49	0.53	**0.68**	0.38	0.53
DLpTCR	0.57	0.66	0.43	0.63	0.57	0.45	0.56	0.50	**0.72**	0.67	0.44	0.38	0.53	0.45	0.50	**0.63**	0.53
epiTCR	0.52	0.59	0.56	0.53	0.55	0.49	0.47	0.47	0.53	**0.74**	0.56	0.54	0.54	0.48	0.47	0.49	0.48
ERGO	0.62	0.68	0.67	0.45	0.60	0.50	0.54	0.49	0.55	0.63	0.68	0.53	0.56	**0.67**	0.63	0.46	**0.59**
ERGO-II	0.73	0.76	0.79	0.65	0.73	0.44	0.54	0.54	0.49	0.63	0.50	**0.61**	0.54	0.48	0.52	**0.62**	0.54
ImRex	0.45	0.77	**0.88**	0.37	0.62	**0.58**	0.53	0.47	0.43	0.47	0.59	**0.78**	0.55	0.56	0.44	**0.61**	0.54
iTCep	0.48	0.67	0.64	0.81	0.65	0.47	0.47	0.51	0.43	0.38	**0.68**	0.46	0.49	0.57	0.60	0.52	**0.56**
**MixTCRpred**	0.68	**0.86**	**0.92**	0.96	**0.85**	-	0.53	**0.70**	0.56	-	0.66	-	**0.56**	-	-	-	-
NetTCR-2.2	0.35	0.82	0.86	0.93	0.74	0.53	**0.56**	0.49	**0.63**	**0.74**	0.52	0.50	**0.57**	**0.65**	**0.64**	0.37	0.56
**NetTCR-Cat**	-	**0.87**	**0.96**	0.93	**0.82**	-	-	-	**0.61**	-	-	-	0.52	-	-	-	-
PanPep	0.55	0.41	0.65	0.43	0.51	0.38	0.43	0.53	0.47	0.45	**0.70**	0.40	0.48	0.50	0.60	0.39	0.50
pMTnet	0.46	0.71	0.54	0.54	0.57	0.51	0.54	0.51	0.49	0.49	0.50	0.58	0.52	0.56	0.55	0.49	0.53
STAPLER	0.61	**0.84**	0.87	0.93	0.81	0.49	0.53	0.51	0.52	0.37	0.65	0.58	0.52	0.47	0.50	0.52	0.50
TCellMatch	0.60	0.61	0.64	0.51	0.59	**0.56**	0.52	0.49	0.46	**0.72**	0.42	0.49	0.52	0.56	0.39	0.49	0.48
**TCRGP**	0.64	0.77	0.76	**0.97**	0.78	-	-	-	-	-	-	-	-	-	-	-	-
TEIM	0.52	0.73	0.65	**0.98**	0.72	0.38	0.47	0.51	0.41	0.71	0.62	0.45	0.51	0.46	0.61	0.27	0.45
TEINet	**0.85**	0.76	0.49	**0.96**	0.76	0.49	**0.58**	**0.60**	0.46	0.45	0.68	0.56	0.55	**0.64**	**0.64**	0.40	**0.56**
TITAN	0.60	0.82	0.24	0.91	0.64	**0.55**	0.54	0.58	0.58	0.62	0.50	**0.60**	**0.57**	0.62	0.57	0.44	0.54
TULIP-TCR	**0.95**	0.79	0.65	0.95	**0.84**	0.50	**0.60**	**0.62**	0.47	0.51	**0.68**	0.41	0.54	0.62	0.55	0.43	0.53

Table S3 | Performance on the viral dataset per epitope, related to Figure 2. The reported values correspond to the performance evaluated through the AUC (Area under the receiver operating characteristic curve) per epitope for classification grouped by the occurrence of CDR3β sequences in public datbases into High ($n \geq 500$), Medium ($n \geq 4$), and Low ($n < 4$). Categorical models are marked with boldface method names. The average indicates the mean of all epitopes within the corresponding group assuming a score of 0.5 when no model was provided for this epitope. The metric scores of the three best models are highlighted in boldface for each metric. The epitopes are abbreviated by their first three peptide residues.

	Full Data<br>AUC	APS	F1-Score	Pearson	Spearman	Per TCR<br>AUC	APS	F1-Score	Pearson	Spearman
ATM-TCR	0.50	0.31	**0.48**	**0.07**	-0.05	0.53±0.10	**0.34±0.17**	0.37±0.22	0.06±0.16	0.04±0.16
AttnTAP	0.47	0.29	0.47	-0.04	-0.03	0.46±0.11	0.29±0.17	0.42±0.19	-0.05±0.18	-0.03±0.19
BERTrand	0.45	0.27	0.47	-0.08	-0.09	0.43±0.10	0.26±0.16	0.42±0.19	-0.12±0.16	-0.16±0.15
DLpTCR	0.54	0.35	0.47	0.06	**0.10**	0.51±0.09	0.29±0.16	0.31±0.27	0.03±0.15	0.01±0.16
epiTCR	0.53	0.32	0.47	0.04	**0.10**	0.47±0.11	0.29±0.17	0.41±0.19	-0.02±0.13	0.03±0.11
ERGO	0.53	0.32	**0.48**	0.03	0.02	0.44±0.15	0.29±0.14	0.40±0.21	-0.17±0.21	-0.14±0.19
ERGO-II	**0.54**	**0.37**	**0.48**	0.03	-0.01	0.51±0.09	0.32±0.19	0.28±0.29	-0.01±0.20	-0.03±0.17
ImRex	0.53	**0.36**	0.48	0.07	0.05	0.56±0.14	0.34±0.20	**0.42±0.19**	**0.09±0.18**	0.09±0.18
iTCep	**0.56**	**0.38**	0.47	**0.09**	**0.11**	**0.61±0.15**	**0.39±0.19**	**0.42±0.19**	**0.09±0.19**	**0.21±0.22**
NetTCR-2.2	0.43	0.28	0.47	-0.13	-0.16	0.51±0.13	0.31±0.14	0.42±0.19	-0.04±0.19	-0.07±0.18
PanPep	0.48	0.30	0.47	-0.03	-0.04	0.49±0.08	0.31±0.18	0.41±0.19	-0.01±0.09	-0.00±0.08
pMTnet	**0.54**	0.34	0.47	**0.09**	0.09	0.48±0.10	0.30±0.16	0.41±0.19	-0.03±0.13	-0.06±0.16
STAPLER	0.47	0.32	0.47	-0.02	-0.10	0.49±0.10	0.30±0.18	0.42±0.19	-0.00±0.11	-0.02±0.17
TCellMatch	0.54	0.32	0.47	0.07	0.09	0.53±0.07	0.29±0.16	0.42±0.17	0.07±0.08	0.06±0.14
TEIM	0.48	0.29	0.47	-0.03	-0.02	0.55±0.09	0.33±0.18	0.38±0.22	0.05±0.17	0.04±0.16
TEINet	0.52	0.33	0.47	0.04	0.03	0.47±0.09	0.29±0.14	0.41±0.19	-0.07±0.15	-0.06±0.13
TITAN	0.52	0.32	0.47	0.01	-0.00	**0.57±0.10**	0.33±0.16	0.42±0.19	0.08±0.15	**0.09±0.14**
TULIP-TCR	0.53	0.34	0.48	0.05	0.01	**0.59±0.11**	**0.40±0.14**	**0.42±0.18**	**0.16±0.18**	**0.12±0.17**

Table S4 | Performance on the mutation dataset, related to Figure 3. The reported values correspond to the performance evaluated on either the full dataset or per TCR for classification (AUC: Area under the receiver operating characteristic curve, APS: average precision score, F1-Score) or regression (Pearson and Spearman correlation coefficient). The mean performance and standard deviation are indicated when the performance was evaluated per TCR. The three best scores for each metric are highlighted in boldface.

	Per CMV TCR<br>AUC	APS	F1-Score	Pearson	Spearman	Per Tumor TCR<br>AUC	APS	F1-Score	Pearson	Spearman
ATM-TCR	0.54±0.10	0.33±0.16	0.33±0.22	0.06±0.16	0.05±0.17	0.50±0.08	0.37±0.19	0.51±0.19	0.07±0.17	-0.01±0.15
AttnTAP	0.44±0.08	0.25±0.13	0.39±0.19	-0.10±0.13	-0.08±0.16	**0.56±0.14**	0.43±0.23	**0.51±0.19**	**0.12±0.24**	0.11±0.24
BERTrand	0.43±0.07	0.24±0.15	0.39±0.18	-0.13±0.12	-0.18±0.12	0.45±0.17	0.34±0.18	0.51±0.19	-0.10±0.27	-0.10±0.23
DLpTCR	0.52±0.08	0.27±0.15	0.25±0.26	0.01±0.12	-0.03±0.13	0.50±0.11	0.36±0.20	0.51±0.19	0.09±0.22	**0.14±0.21**
epiTCR	0.45±0.10	0.26±0.15	0.39±0.19	-0.04±0.11	-0.00±0.10	0.53±0.14	0.39±0.18	0.51±0.18	0.04±0.20	0.14±0.10
ERGO	0.44±0.16	0.28±0.13	0.39±0.19	-0.19±0.22	-0.15±0.18	0.43±0.12	0.32±0.18	0.46±0.29	-0.12±0.17	-0.13±0.22
ERGO-II	0.49±0.09	0.27±0.17	0.21±0.28	-0.01±0.21	-0.03±0.19	0.55±0.06	**0.46±0.21**	0.51±0.19	0.00±0.17	-0.03±0.13
ImRex	0.53±0.11	0.28±0.15	**0.39±0.19**	0.03±0.11	0.03±0.12	**0.67±0.17**	**0.53±0.23**	**0.52±0.20**	**0.30±0.23**	**0.30±0.21**
iTCep	**0.63±0.14**	**0.37±0.18**	**0.39±0.19**	0.10±0.20	**0.24±0.19**	0.56±0.19	**0.45±0.24**	0.51±0.18	0.09±0.15	0.08±0.27
NetTCR-2.2	0.57±0.07	0.32±0.15	0.39±0.19	0.05±0.05	0.02±0.07	0.32±0.03	0.28±0.13	0.51±0.18	-0.35±0.10	-0.37±0.07
PanPep	0.50±0.08	0.29±0.17	0.39±0.19	0.01±0.09	0.01±0.07	0.47±0.07	0.35±0.20	0.51±0.18	-0.04±0.08	-0.06±0.09
pMTnet	0.48±0.11	0.28±0.17	0.39±0.18	-0.02±0.13	-0.05±0.17	0.47±0.06	0.37±0.12	0.51±0.18	-0.06±0.15	-0.06±0.13
STAPLER	0.47±0.09	0.26±0.15	0.39±0.19	-0.02±0.08	-0.03±0.15	0.56±0.14	0.43±0.21	**0.52±0.19**	0.05±0.16	0.01±0.24
TCellMatch	0.51±0.07	0.25±0.14	0.39±0.17	0.06±0.08	0.00±0.10	**0.59±0.05**	0.43±0.17	0.51±0.14	**0.11±0.07**	**0.23±0.08**
TEIM	**0.59±0.06**	**0.34±0.18**	0.34±0.22	**0.12±0.09**	0.10±0.09	0.44±0.10	0.32±0.17	0.51±0.19	-0.17±0.21	-0.14±0.21
TEINet	0.47±0.10	0.26±0.13	0.39±0.19	-0.09±0.16	-0.08±0.13	0.48±0.05	0.37±0.15	0.50±0.19	0.00±0.07	-0.02±0.10
TITAN	0.58±0.09	0.31±0.15	0.39±0.19	**0.12±0.12**	**0.12±0.12**	0.52±0.12	0.38±0.20	0.51±0.18	-0.05±0.19	0.01±0.18
TULIP-TCR	**0.63±0.09**	**0.43±0.13**	**0.40±0.18**	**0.24±0.10**	**0.19±0.10**	0.46±0.08	0.33±0.16	0.51±0.19	-0.09±0.15	-0.11±0.13

Table S5 | Separate performance on the two subsets of the mutation dataset, related to Figure 3. The reported values correspond to the performance evaluated per TCR for classification (AUC: Area under the receiver operating characteristic curve, APS: average precision score, F1-Score) or regression (Pearson and Spearman correlation coefficient) calculated over CMV or Tumor TCRs. The mean performance and standard deviation are indicated when the performance was evaluated per TCR. The three best scores for each metric are highlighted in boldface.

	Full Data AUC	APS	F1-Score	Pearson	Spearman	Per TCR AUC	APS	F1-Score	Pearson	Spearman
ATM-TCR	**0.58**	**0.49**	0.59	-0.05	**0.17**	**0.58±0.16**	**0.47±0.27**	0.54±0.27	0.06±0.12	**0.10±0.17**
AttnTAP	0.45	0.40	0.59	-0.01	-0.08	0.42±0.10	0.41±0.24	0.54±0.27	-0.05±0.15	-0.15±0.15
BERTrand	**0.56**	**0.47**	**0.59**	**0.13**	**0.13**	0.51±0.08	0.44±0.24	0.54±0.27	0.04±0.07	0.05±0.07
epiTCR	0.52	0.42	0.59	0.00	-0.01	0.53±0.08	0.45±0.24	0.54±0.27	0.04±0.12	0.06±0.09
ERGO	0.45	0.39	**0.59**	-0.13	-0.10	0.50±0.16	0.46±0.24	**0.54±0.27**	**0.06±0.21**	0.08±0.21
ERGO-II	0.52	0.44	0.59	**0.15**	0.10	**0.58±0.09**	**0.49±0.25**	**0.54±0.27**	**0.15±0.09**	**0.12±0.09**
ImRex	0.43	0.36	0.59	-0.16	-0.15	0.46±0.10	0.42±0.23	0.54±0.27	-0.07±0.21	-0.09±0.20
iTCep	0.45	0.37	**0.60**	-0.15	-0.11	0.45±0.13	0.39±0.21	0.54±0.27	-0.15±0.08	-0.13±0.11
PanPep	0.48	0.40	0.59	-0.05	-0.06	0.49±0.09	0.42±0.25	0.54±0.27	-0.04±0.09	-0.05±0.11
TCellMatch	0.44	0.40	0.59	-0.19	-0.10	0.42±0.07	0.40±0.24	0.54±0.27	-0.24±0.13	-0.16±0.11
TEIM	0.38	0.35	0.59	-0.18	-0.21	0.43±0.12	0.40±0.25	0.54±0.27	-0.12±0.16	-0.11±0.14
TEINet	0.50	0.42	0.59	0.01	-0.02	0.46±0.09	0.42±0.23	0.54±0.27	-0.01±0.10	-0.07±0.11
TITAN	0.49	0.41	0.59	-0.03	-0.03	0.50±0.12	0.41±0.24	0.54±0.27	-0.05±0.11	-0.04±0.10
TULIP-TCR	**0.57**	**0.50**	0.59	**0.13**	**0.16**	**0.56±0.11**	**0.50±0.26**	**0.54±0.27**	**0.12±0.16**	**0.15±0.16**

Table S6 | Performance of applicable methods on the murine mutation dataset, related to Figure 3. The reported values correspond to the performance evaluated on either the full dataset or per TCR for classification (AUC: Area under the receiver operating characteristic curve, APS: average precision score, F1-Score) or regression (Pearson and Spearman correlation coefficient). The mean performance and standard deviation are indicated when the performance was evaluated per TCR. The three best scores for each metric are highlighted in boldface.
